# Crystal structure and functional analysis of human C1ORF123

**DOI:** 10.7717/peerj.5377

**Published:** 2018-09-28

**Authors:** Siti Nurulnabila A. Rahaman, Jastina Mat Yusop, Zeti-Azura Mohamed-Hussein, Wan Mohd Aizat, Kok Lian Ho, Aik-Hong Teh, Jitka Waterman, Boon Keat Tan, Hwei Ling Tan, Adelicia Yongling Li, Ee Sin Chen, Chyan Leong Ng

**Affiliations:** 1 Institute of Systems Biology, Universiti Kebangsaan Malaysia, Bangi, Selangor, Malaysia; 2 Center for Frontier Sciences, Faculty of Science and Technology, Universiti Kebangsaan Malaysia, Bangi, Selangor, Malaysia; 3 Department of Pathology, Faculty of Medicine and Health Sciences, Universiti Putra Malaysia, Serdang, Selangor, Malaysia; 4 Centre for Chemical Biology, Universiti Sains Malaysia, Bayan Lepas, Penang, Malaysia; 5 Diamond Light Source, Harwell Science and Innovation Campus, Didcot, England, United Kingdom; 6 Division of Human Biology, School of Medicine, International Medical University, Bukit Jalil, Kuala Lumpur, Malaysia; 7 Department of Biochemistry, Yong Loo Lin School of Medicine, National University of Singapore, Singapore

**Keywords:** C1ORF123, DUF866, Internal symmetry, Zinc-binding domain, Mitochondrial oxidative phosphorylation, Crystal structure, CXXC motif

## Abstract

Proteins of the DUF866 superfamily are exclusively found in eukaryotic cells. A member of the DUF866 superfamily, C1ORF123, is a human protein found in the open reading frame 123 of chromosome 1. The physiological role of C1ORF123 is yet to be determined. The only available protein structure of the DUF866 family shares just 26% sequence similarity and does not contain a zinc binding motif. Here, we present the crystal structure of the recombinant human C1ORF123 protein (rC1ORF123). The structure has a 2-fold internal symmetry dividing the monomeric protein into two mirrored halves that comprise of distinct electrostatic potential. The N-terminal half of rC1ORF123 includes a zinc-binding domain interacting with a zinc ion near to a potential ligand binding cavity. Functional studies of human C1ORF123 and its homologue in the fission yeast *Schizosaccharomyces pombe* (SpEss1) point to a role of DUF866 protein in mitochondrial oxidative phosphorylation.

## Introduction

The human C1ORF123 protein belongs to the eukaryotic protein superfamily DUF866 (PF05907), with unknown function. To date, 861 sequences of PF05907 proteins have been found in 735 eukaryotes (http://pfam.xfam.org/family/PF05907) including fungi, apicomplexa, plants and mammals. Most of these genes encode for the proteins with approximately 160 amino acids (Finn et al., 2015). The yeast homologue was identified as a non-essential gene of unknown physiological function (Huh et al., 2003). Currently, the only structure that is available from the DUF866 protein superfamily is the hypothetical protein MAL13P1.257 from protozoan parasite *Plasmodium falciparum* that causes malaria in humans (Holmes et al., 2006). The protein structure of MAL13P1.257 consists of a single domain with a novel fold and likely forms a weak biological dimer. The DUF866 protein family has been proposed to contain several conserved regions across the proteins in the superfamily. Two conserved CXXC motifs are present in most of the proteins in the superfamily including those of human origin, however are absent in MAL13P1.257 from *P. falciparum* (Holmes et al., 2006). This suggests that the biological function of DUF866 proteins may have diverged through evolution events. The two CXXC motifs were predicted to play a role of a zinc-binding motif (Passerini et al., 2007), hence C1ORF123 and other DUF866 proteins are likely to be metalloproteins. Nonetheless, structures of the DUF866 proteins with the CXXC motifs, that may shed light on the functional understanding of this eukaryotic conserved superfamily of proteins, are yet to be revealed.

Human C1ORF123 is encoded by an open reading frame that results from the splicing of 8 exons (http://asia.ensembl.org/), which consist of 160 amino acids (NP_060357.1). However, two isoforms of C1ORF123 transcript (NP_001291688.1 and NP_001291689.1) that encodes proteins consisting of 143 (isoform 2) and 113 amino acids (isoform 3) respectively, have also been identified. The isoforms lack one and two alternate in-frame exons at the 5′ end, respectively. The C1ORF123 transcripts have been found in a range of tissues and organs (Su et al., 2004).

Recently, the advancement of genomic, transcriptomic, proteomic studies have provided data for functional understanding of the DUF866 proteins. For instance, human C1ORF123 was found to have a significantly high number of transcripts in the oocytes of Polycystic Ovarian Syndrome (PCOS) patients (Wood et al., 2006). People with schizophrenia and bipolar disorder have a high expression of C1ORF123 in their hippocampus (Schubert, Föcking & Cotter, 2015). The C1ORF123 homologue in rat was found in the frontal cortex of aged rats with slow wave sleep (Vazquez, Hall & Greco, 2009), and was overexpressed in the prefrontal cortex of methamphetamine-treated rats. These findings suggest that the DUF866 protein family may play a role in the psychotic disorders and brain function (Wearne et al., 2014). Consistent with its neurological link in mammals, C1ORF123 homologue has also been found in the electric organ of the electric ray *Torpedo californica* together with proteins that are related to neuromuscular junctions and presynapsis, suggesting its function in synapse structure and maintenance (Mate et al., 2011; Mate et al., 2012). While the C1ORF123 homologue in goat was identified as an adipokine that may be involved in the endocrine function (Restelli et al., 2014), C1ORF123 has been identified as one of the human O-GlcNAc transferase (OGT) interactors, suggesting its role in post translational modification (Deng et al., 2014).

In this article, we report the crystal structure of full-length human C1ORF123 (rC1ORF123) that contains a 2-fold internal symmetry, which divides the monomer protein into two halves. Distinct electrostatic surface potentials on each half suggest functional evolution via gene duplication. The two CXXC motifs (*CX*_2_*CX*_30_*CX*_2_*C*) form a zinc-binding domain, which binds a zinc ion. This is similar to the C-terminal domain of human RIG-I-like receptor LGP2. A cavity that is located near to the zinc-binding motif undergoes a conformational change upon glycerol molecule binding, suggesting a functional role of C1ORF123 protein upon ligand interaction. Functional studies of rC1ORF123 and its counterpart from *Schizosaccharomyces pombe* shed a light on its potential role in mitochondrial oxidative phosphorylation.

## Material and Methods

### Multiple Sequences alignment and phylogenetic tree analysis

Multiple sequence alignment (MSA) was conducted using CLUSTALX (Larkin et al., 2007) for 499 selected members in DUF866 family (about 60% of the family) that contain DUF866 standalone domain consisting of 150–170 amino acids. The output of the alignment was used to construct a maximum likelihood phylogenetic tree with MEGA 7 (Kumar, Stecher & Tamura, 2016) using maximum likelihood distances. Bootstrap values from the 1,000 replicates were used to assess the robustness of the tree.

MSA analysis of a simplified subset was also conducted using Clustal Omega (Sievers et al., 2011). This subset comprised for 21 homologues of C1ORF123 that include primates (chimpanzee [gi—332809033—] and monkey [gi—109004854—]), rodents (house mouse [gi—21539639—] and rat [gi—77627996—]), placental mammals (cow [gi—84000233—] and dog [gi—345800183—]), chicken [gi—50751624—], zebrafish [gi—192455700—], frog [gi—301603650—], fruit fly [gi—24653302—], mosquito [gi—158298171—], round worm (*Caenorhabditis elegans* [gi—392920054—]), plants (*Arabidopsis thaliana* [gi—18418118—] and rice [gi—297597036—]), fungi (Red mold [gi—85110386—], rice fungus [gi—389623525—], *Saccharomyces cerevisiae* [gi—6319933], *Schizosaccharomyces pombe* [gi—162312271—], *Kluyveromyces lactis* [gi—50311037—] and *Eremothecium gossypii* [gi—302309171—]) and *Plasmodium falciparum* [gi—124513736l].

### Crystal structure determination and refinement

The structure of rC1ORF123 was determined using the automated molecular replacement platform Balbes (Long et al., 2008) with the X-ray diffraction data collected and processed as previously reported (Rahaman et al., 2016). The structure of MAL13P1.257 from *P. falciparum* (Protein Data Bank code: 1ZSO), which shares 26% of sequence identity, was used as a search model. The molecular replacement solution found two molecules of rC1ORF123 protein in the asymmetric unit. The initial molecular replacement model was subjected to initial automated model building using ARP/WARP (Langer et al., 2008) followed by manual model building in COOT (Emsley et al., 2010). The restrained refinement which included the TLS refinement was performed with REFMAC (Murshudov et al., 2011). The final structure with two well-ordered full length C1ORF123 (residue 1–160) molecules was refined to *R*_work_ of 0.1754 and *R*_free_ 0.2203 with all backbone dihedral angles falling into the most favoured or allowed regions of the Ramachandran plot, as defined by Molprobity (Chen et al., 2010). The structure refinement statistics are summarized in Table 1.

**Table 1:** Crystallographic data and refinement statistics for rC1ORF123.

	**rC1ORF123**
Resolution range (Å)	30.90–1.90 (1.94–1.90)^a^
Space group	*P*2_1_2_1_2_1_
Unit cell	
a, b, c (Å)	59.32 65.35 95.05
*α*, β, *γ* (°)	90.0
Refinement statistics	
R_cryst_, R_free_ (%)	17.56, 22.04
No. of molecules per asymmetric unit	2
No. of water molecules	179
No. of glycerol molecules	6
No. of β-mercaptoethanol molecules	1
No. of Zinc ion	2
No. of Cloride ion	2
Root mean square deviation from ideal values (r.m.s.d.)	
Bond length (Å)	0.0205
Bond angle (^∘^)	2.0312
Ramachandran plot statistics	
Favoured regions (%)	95.51
Allowed regions (%)	3.85
Average B factors	
Monomer A and B	31.89

**Notes.**

a The parentheses indicate the values for the highest resolution shell.

### Structural and functional analysis using bioinformatics tools

The structure of rC1ORF123 was analyzed with a portfolio of bioinformatics tools. The DALI server (http://ekhidna2.biocenter.helsinki.fi/dali/oldstyle.html) was used for 3D structure comparison (Holm & Rosenström, 2010). The jsPISA (http://www.ccp4.ac.uk/pisa/) was used for protein interface analysis (Krissinel, 2015). CE-Symm and SymD (https://symd.nci.nih.gov/) were used to calculate the internal symmetry of C1ORF123 (Myers-Turnbull et al., 2014; Tai et al., 2014). The program COACH (https://zhanglab.ccmb.med.umich.edu/COACH/) was used for the prediction of the protein-ligand binding site (Yang, Roy & Zhang, 2013). Computed Atlas of Surface Topography of proteins (CASTp) (http://sts.bioe.uic.edu/castp/index.html?2r7g) was used to analyze the cavity and surface pocket of a protein (Dundas et al., 2006). Phyre2 (http://www.sbg.bio.ic.ac.uk/phyre2/html/page.cgi?id=index) (Kelley et al., 2015) was used for a 3D structure prediction of DUF866 domain of carbon-nitrogen protein G0QVS7 from *Ichthyophthirius multifiliis*, while I-Tasser (https://zhanglab.ccmb.med.umich.edu/I-TASSER/) (Zhang, 2008) was used to obtain the model structure of isoform 2 and 3 of C1ORF123. The homology model of spEss1 was generated using SwissModel (https://swissmodel.expasy.org/) (Benkert, Biasini & Schwede, 2010) with C1ORF123 as a template.

### Total Protein Extraction of human HeLa cells

Cryopreserved human HeLa cell line (ATCC^^®^^ CCL-2^™^) was prepared and maintained in the 85% Dulbecco’s Modified Eagle’s Medium (DMEM) supplemented with 10% (v/v) FBS and 5% DMSO. Total protein extraction was performed as directed in the Pierce Magnetic Protein A/G Immunoprecipitation kit (Thermo Fisher Scientific, Waltham, MA, USA). 2 mL of cryopreserved HeLa cell (containing ∼2 × 10^6^ of cells) were thawed and centrifuged at 1,000× g for 5 min. Supernatant was removed and the remaining pellet was washed in pH 7.4 phosphate saline buffer, followed by centrifugation for 5 min at 1,000× g. Proteins in the pellet were extracted in the ice-cold lysis buffer (0.025 M Tris, 0.15M NaCl, 0.001M EDTA, 1% NP40, 5% glycerol, 1X protease inhibitor). The volume of lysis buffer was added based on the ratio to the wet cell pellet which is 10:1 (v/w). The lysate was incubated for 10 min with mixing followed by centrifugation at ∼13,000× g for 10 min to remove the cell debris. The total protein content was measured using the NanoDrop 1000 V3.7 spectrophotometer (Thermo Scientific, Waltham, MA, USA).

### Immunoprecipitation of C1ORF123 using rabbit anti-C1ORF123 IgG

Immunoprecipitations (IP) were performed using rabbit anti-C1ORF123 IgG (Sigma-Aldrich, St. Louis, MO, USA) against antigens of HeLa cell lysates to form antigen/antibody complexes in binding buffer (0.025M Tris, 0.15M NaCl, 0.001M EDTA, 1% NP40, 5% glycerol). The anti-C1ORF123 antibody was first verified to react with rC1ORF123 by Western blot followed by the IP method that applied in this study. Protein A/G magnetic beads that treated with anti-C1ORF123 was incubated with rC1ORF123 followed by Pierce Protein Magnetic A/G Immunoprecipitation (Thermo Fisher Scientific, Waltham, MA, USA) method. Eluate that contains rC1ORF123 protein was further confirmed by anti-PentaHis-HRP using Western Blot (Qiagen, Valencia, CA, USA).

To obtain endogenous C1ORF123 and its interacting partners, the verified IP method was applied with 6 µg of anti-C1ORF123 added to HeLa cell lysate sample prepared as above. Additionally, another HeLa cell lysate was added with 60 µg of the purified rC1ORF123 protein to enrich interacting proteins. To identify the false positive proteins that unspecifically bound to the rabbit IgG, the IgG rabbit polyclonal (Abcam, Cambridge, UK) was incubated with HeLa cell lysate instead of anti-C1ORF123. All mixtures were incubated overnight at 4 °C. Pre-equilibrated protein A/G magnetic beads were added and incubated at room temperature with gentle rolling for 1 hour. The non-specifically bound proteins were removed by washing steps, and the potential C1ORF123 protein partners were eluted from the magnetic beads particles using elution buffer (glycine pH 2). The eluate was then neutralized by Tris pH 8.5. The IP reaction for control experiment were performed in five replicates before the sample with anti-C1ORF123 and HeLa cell lysate (antiC1_HeLa), and sample with rC1ORF123 added to the anti-C1ORF123 and HeLa cell lysate (antiC1_rC1_HeLa ) were performed.

All the immunoprecipitated samples were analyzed using high resolution Mass spectrometry with nano Liquid Chromatography Orbitrap Mass Analyzer (Dionex 3000 Ultimate RSLCnano/ Orbitrap fusion) based on the manufacturer provided protocol (LTQ-Orbitrap XL; Thermo Fisher Scientific) with slight modification. In short, samples were loaded in EASY-Spray Column Acclaim PepMap C18 100 Å column for 100 min with reverse phase gradient; 5–40% of solvent B (containing 0.1% formic acid in acetonitrile) for 91 min, 2 min to 95% of solvent B, and 6 min at 95% of solvent B, back to 5% of solvent B in 2 min at a flow rate of 300 nL/min. The analyzed MS/MS data from orbitrap MS(OTMS) were carried out using the Thermo Scientific™ Proteome Discoverer™ Software Version 2.0 against the *Homo sapiens* UNIPROT database for protein identification (Giansanti et al., 2016). All peptides were validated using the Percolator^^®^^ algorithm, based on *q*-value at a 1% false discovery rate (FDR). Only proteins with number of peptides more than 2 were identified and selected from the sample. Venn diagram was used to group proteins identified from control (appeared in at least two replicates), antiC1_HeLa and antiC1_rC1_HeLa samples. The C1ORF123 interacting protein partners were identified for protein that found in both antiC1_HeLa and antiC1_rC1_HeLa samples but not in the control.

### Phenotype characterization in *Schizosaccharomyces pombe*

*Schizosaccharomyces pombe* SPBC2D10.03c/Ess1 was identified to be the counterpart of human C1orf123 by Pombase and NCBI BLASTp. The null mutant was obtained from Bioneer (ver 2.0) haploid gene deletion library (Bionee, Daejeon, South Korea) and a strain without nutritional markers was created by crossing with prototrophic wild-type (WT) 972 strain. PCR with locus specific primers were performed to confirm the gene deletion. Previously published procedures to culture and test drug hypersensitivity in fission yeast were followed (Tay et al., 2013; Nguyen et al., 2016). Briefly, cells were grown in YEA (3% glucose, 0.5% yeast extract, 75 mg/ml L-adenine) to log-phase, ten-fold serial-diluted and spotted onto media agar plates incorporated with drugs: hydrogen peroxide (H_2_O_2_), hydroxyurea (HU) (Sigma-Aldrich) and doxorubicin (Wako Pure Chemical Industries Ltd, Japan). Cell growth was documented 3 and 6 days after spotting.

## Results

### Multiple sequence alignment and phylogenetic analysis of DUF866

**Figure 1:**
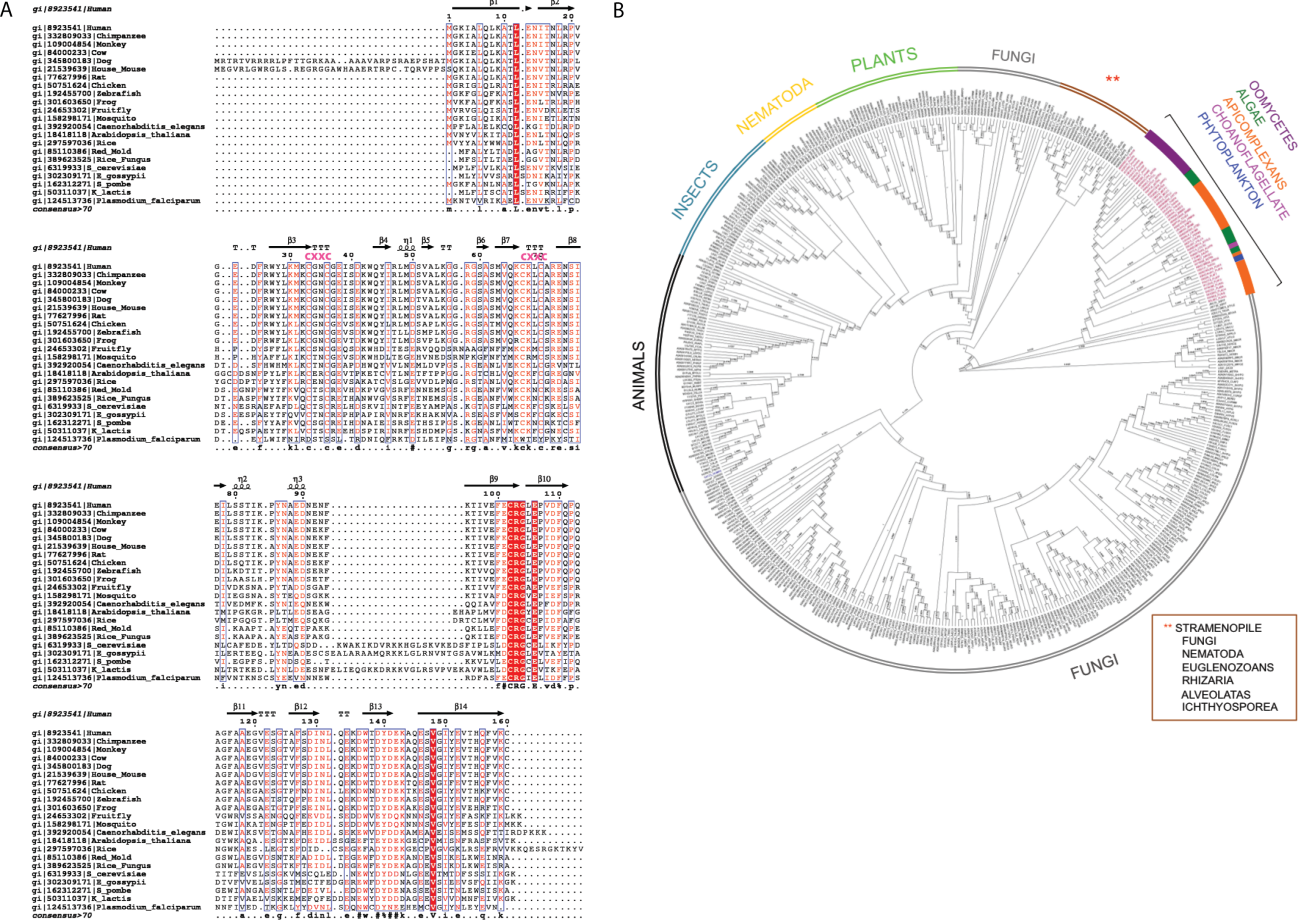
Multiple sequence alignment and phylogenetic analysis of DUF866 proteins. (A) Human C1ORF123 protein was aligned with 21 homologues including primates (chimpanzee [gi—332809033—] and monkey [gi—109004854—]), rodents (house mouse [gi—21539639—] and rat [gi—77627996—]), placental mammals (cow [gi—84000233—] and dog [gi—345800183—]), chicken [gi—50751624—], zebrafish [gi—192455700—], frog [gi—301603650—], fruit fly [gi—24653302—], mosquito [gi—158298171—], round worm (*Caenorhabditis elegans* [gi—392920054—]), plants (*Arabidopsis thaliana* [gi—18418118—] and rice [gi—297597036—]), fungi (Red mold [gi—85110386—], rice fungus [gi—389623525—], *Saccharomyces cerevisiae* [gi—6319933], *Schizosaccharomyces pombe* [gi—162312271—], *Kluyveromyces lactis* [gi—50311037—] and *Eremothecium gossypii* [gi—302309171—]) and *Plasmodium falciparum* [gi—124513736.The two CXXC motif are indicated in pink. (B) A phylogenetic tree shows evolutionary relationship of 499 members in Pfam DUF866 family that contains single DUF866 domain with a size ranged from 150–170aa. ClustalX was used to align the 499 sequences of DUF866 family proteins (Fig. S1) and MEGA 7 was used to construct the phylogenetic tree using maximum likelihood distances approach. Bootstrap values from 1,000 replicates were used to assess the robustness of the trees. Proteins without the two CXXC motifs (CX_2_CX_30_CX_2_C) are highlighted in red, which include *P. falciparum* MAL13P1.257 while human C1ORF123 (Q9NW4) is in blue.

The multiple sequence alignment showed that human C1ORF123 shares 100% identity with its counterparts in primates (chimpanzee and gorilla), but not in monkey (residue N131D). C1ORF123 also shares a moderate sequence identity (∼40%) with yeast and fungi counterparts including *S. pombe.* However, there is a significant deviation when C1ORF123 is compared with its counterpart in the protozoan parasite *Plasmodium falciparum* (∼26% sequence identity) (Fig. 1A and Fig. S1). Sequence analysis of the DUF866 family members that contain the DUF866 standalone domain with 150-170 residues revealed that 90% of these genes, including C1ORF123, contain two CXXC motifs (CX _2_CX _30_CX _2_C) (Fig. 1B) that are likely to assemble a metal binding site. The remaining 10% of the DUF866 homologues that lack the two CXXC motifs are mainly from apicomplexans (including *P. falciparum*), algae, phytoplankton, oomycetes and choanoflagellate. It is still not known why this subset of organisms does not contain the metal binding motif.

### Overall structure of recombinant human C1ORF123 protein

**Figure 2:**
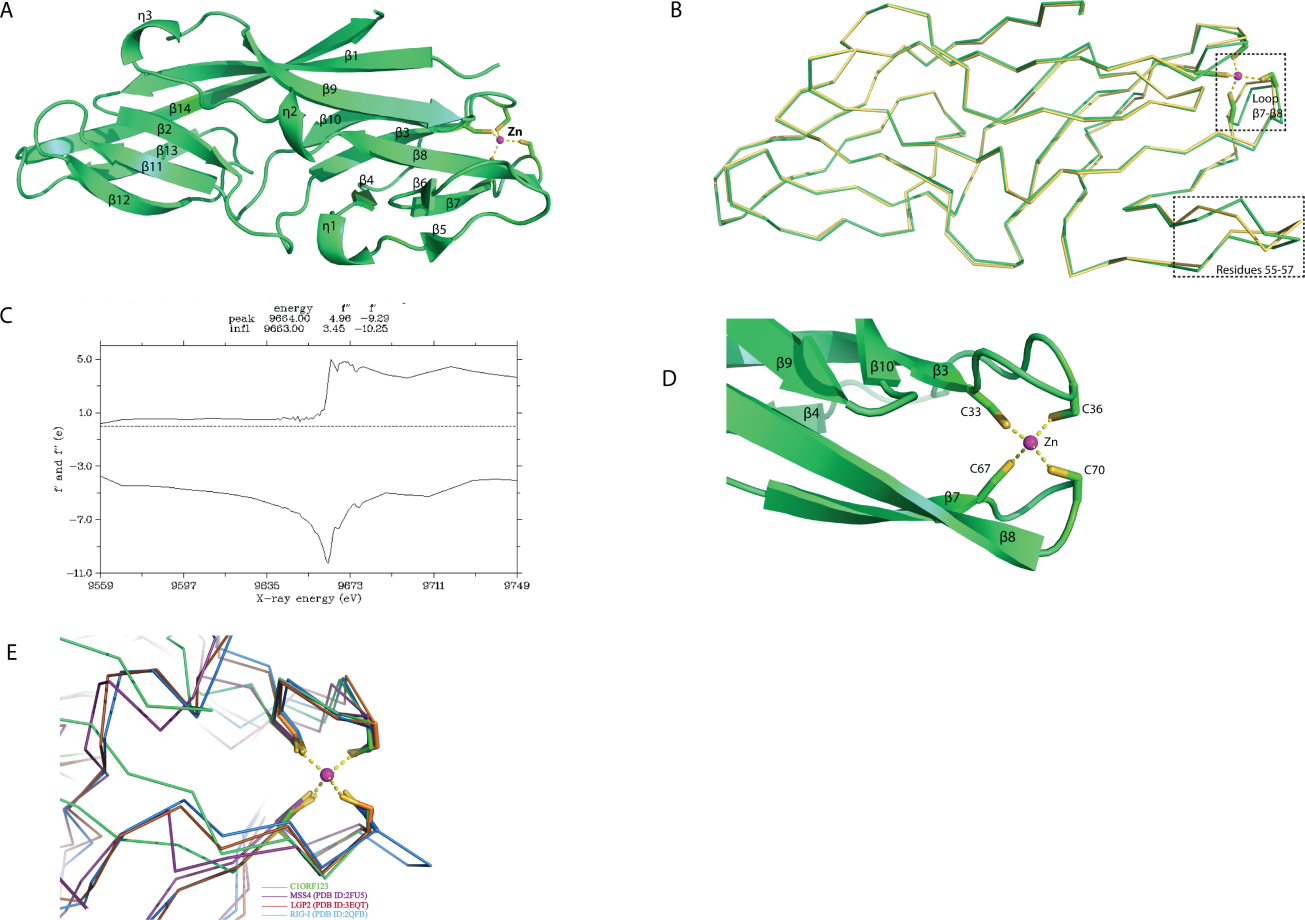
Crystal structure of human C1ORF123 protein. (A) The overall structure of C1ORF123. (B) Superimposition of two C1ORF123 molecules that obtained in the asymmetric (molecule A (green) and molecule B (yellow), the highly deviated region was labeled and boxed. (C) Fluorescence scan for C1ORF123 crystal that shows X-ray absorption edge energy of 9663(eV) that corresponded to K-edge of zinc. (D) The zinc ion is coordinated with four conserved cysteine residues at the N-terminal half of C1ORF123. (E) The structure superimposition of zinc-binding motif of C1ORF123 (Green) and similar motifs were identified in human RIG-I-like receptor LGP2 C-terminal domain (PDB ID: 3EQT, brown), retinoic acid inducible gene 1 protein (RIG-I, PDB ID: 2QFB, blue) and GDP/GTP exchange factor for small Rab-like GTPases)(MSS4, PDB ID: 2FU5, purple).

The overall structure of rC1ORF123 (PDB ID: 5ZRT) reassembled polypeptide chain that assembles fourteen beta strands (β1 to β14) and three small 3_10_ helices (ηA to ηC) as defined by DSSP (Fig. 2A) (Touw et al., 2014). Each molecule of rC1ORF123 has dimensions of approximately 55 × 17 × 17 Å. Structure superimposition using SSM superpose in COOT shows that the two molecules of rC1ORF123 in the asymmetric unit are almost identical with RMSD of 0.45 Å for 160 Cα atoms. The biggest deviation was identified at turn between β5–β6 (residues 55–57) region and at the loop region between β7–β8 (residues 66–72), which contains two cysteine residues that interact with a zinc ion (Fig. 2B). The region of the residues 55–57 in the monomer B are more flexible with weaker electron density observed for the loop compared to the same region in the monomer A, which is stabilized by the interactions with the adjacent symmetry related molecule.

Sequence analysis reveals that C1ORF123 contains a putative metal-binding motif *CX*_2_*CX*_30_*CX*_2_*C* composed of four cysteine residues: C33, C36, C67 and C70. The two CXXC motifs present in C1ORF123 were predicted to bind zinc ion (Passerini et al., 2007). Fluorescence data for elements identification was collected to confirm that the ion that coordinates the two CXXC motifs in the C1ORF123 crystal structure is indeed the zinc ion. The X-ray absorption edge scan at the energy of 9663(eV) that corresponds to the K-edge of zinc confirms the presence of zinc (Fig. 2C). Also, Refmac5 model refinement showed a better fitting of zinc ion to the electron density compared to the iron ion. We conclude that the zinc ion is likely an endogenous ion bound to the recombinant protein during the protein expression in *E. coli* cells because both the protein buffers used during the purification and the crystallization reservoir solution did not contain zinc ions. Hence, a zinc ion interacting with the four-cysteine residues was modeled in each monomer of rC1ORF123 structure in the position bridging the loop β3–β4 and the loop β7–β8 (Fig. 2D).

Protein interface analysis of the crystal structure of rC1ORF123 using PDBePISA (Krissinel, 2015) shows that the two molecules of rC1ORF123 in the asymmetric unit of crystal only have a weak contact with an interface area of 598 Å^2^ (∼7% of a total surface area of rC1ORF123) suggesting that rC1ORF123 is likely not a biological dimer in solution as shown in the SEC analysis that was reported previously (Rahaman et al., 2016).

**Figure 3:**
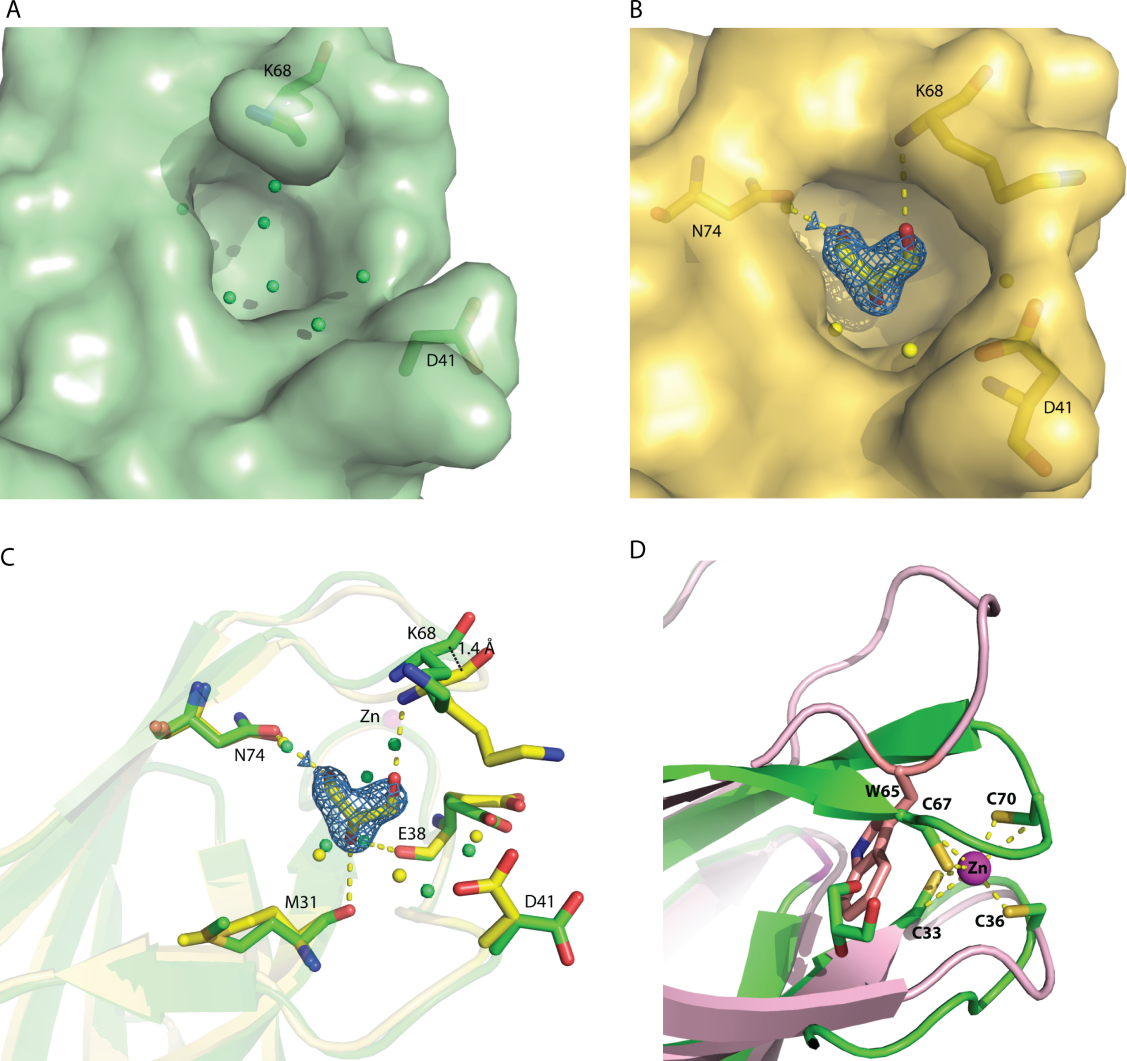
The identified cavity of C1ORF123. (A) The cavity of C1ORF123 that contains water molecules shows in ‘open-form’, (B) The cavity of C1ORF123 that contains a glycerol molecule bound shows in ‘closed-form’. (C) The overall cavity of C1ORF123 with a glycerol molecule interacts with the surrounding residues via hydrogen bonds causes the loop of β7–β8 deviated for 1.4 Å. (D) Superimpose of human C1ORF123 and *Plasmodium falciparum* homologue structure reveal that the cavity of C1ORF123 is unlikely to form in its *P. falciparum* counterpart structure (pink) as a bulky tryptophan side chain was found to occupy the glycerol-binding pocket.

Structural pockets and cavity analysis using Computed Atlas of Surface Topography of Proteins (CASTp) program (Dundas et al., 2006) identified a significant cavity with the pocket size of 242 Å^3^ and 183 Å^3^ for molecule A and B, respectively (Figs. 3A and 3B). This cavity is very different from the one observed in MAL13P1.257*,* which is located at the η1–β5–β6 region (Holmes et al., 2006). In the rC1ORF123 structure, a molecule of glycerol and two water molecules were found in the closed-form cavity of the molecule B while five water molecules were modeled in the open-form cavity of the molecule A.

### Internal domain duplication of C1ORF123

**Figure 4:**
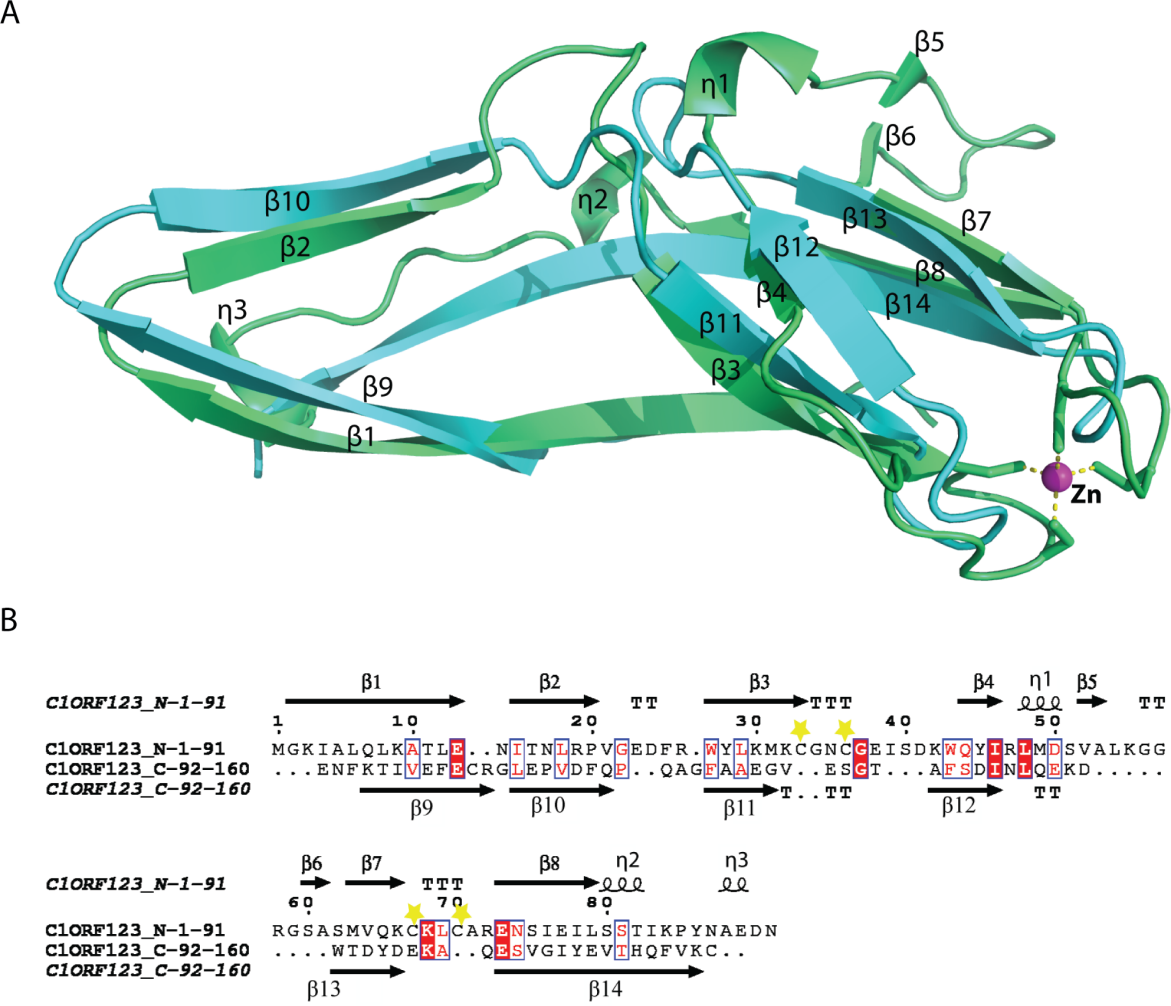
Structure comparison of N-terminal and C-terminal halves of C1ORF123. (A) Superimpose of human C1ORF123 N-terminal half (green) and C-terminal half (cyan). (B) The structural guided sequence alignment of N-terminal and C-terminal of C1ORF123. The conserved four cysteine residues are marked with yellow stars.

A novel fold of the DUF866 family has been previously reported for MAL13P1.257 (Holmes et al., 2006). To our surprise, structure examination of C1ORF123 shows that the N-terminal half (residues 1–91) and C-terminal half (residues 92–160) of C1ORF123 share a very similar fold to each other (Fig. 4A). Structural comparison of the two halves using DALI server shows a *Z*-score of 4.5 with an RMSD of 2.5 Å and most of the secondary structure elements being aligned significantly well (Fig. 4B). Our observation was further supported by the internal symmetry calculation using SymD (Tai et al., 2014) with a *Z*-score of 12.5.

**Figure 5:**
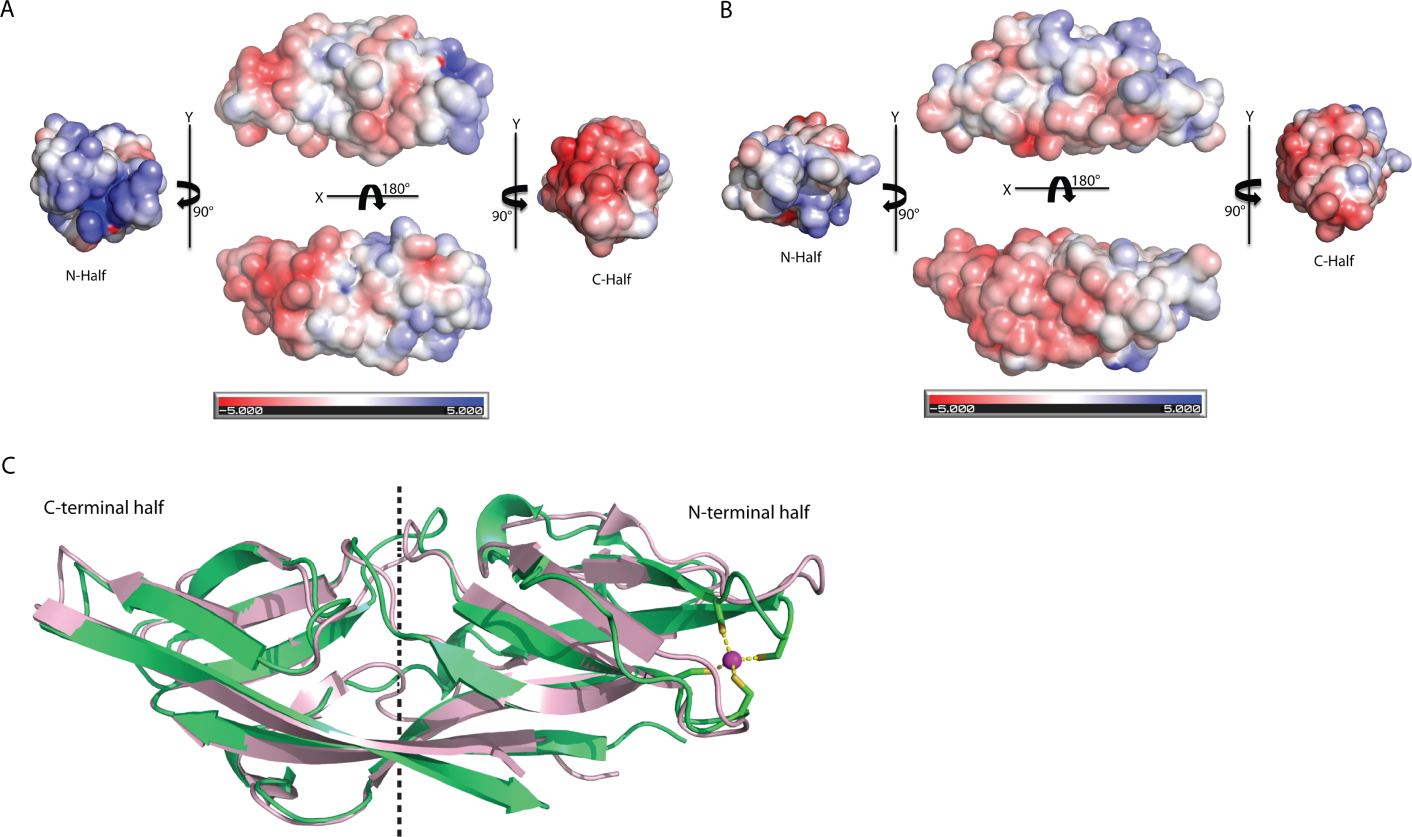
Structure comparison of human C1ORF123 and *Plasmodium falciparum* homologue. (A) Electrostatic potential surface of C1ORF123. (B) Electrostatic potential surface of MAL13P1.257 from *Plasmodium falciparum* generated using CCP4MG (McNicholas et al., 2011). (C) Superimposition of human C1ORF123 (green) and its *Plasmodium falciparum* homologue, MAL13P1.257 protein (pink). The internal two-fold symmetry axis calculated using SymD program is shown as dash line.

The biggest differences between the two halves are (1) the residues 50–61 at the N-terminus half that constitute η1, β5 and β6 have no counterpart in C-terminus half, and is an insertion; (2) η2 is connected to β8 at the N-terminal half compared to its counterpart, a long β14 in the C-terminal half (Fig. 4A). Despite the high structural similarity, sequence alignment between the two halves shows low sequence identity (<10%). Electrostatic potential surface analysis shows that the N-terminal half of C1ORF123 has an overall positively charged surface compared to the C-terminal half that has overall negatively charged surface similar to MAL13P1.257 of *P. falciparum* (Figs. 5A–5B).

Interestingly, structural alignment using DALI (Holm & Rosenström, 2010) also reveals that the C-terminal half of C1ORF123 shares 35% sequence identity (RMSD of 1.1 Å) to its counterpart MAL13P1.257 from *P. falciparum* while the N-terminal half shares only 28% (RMSD of 2.3 Å). This result suggests that the DUF866 proteins are structurally more conserved at the C-terminal half than their N-terminal half.

### Structure comparison of C1ORF123 and its homolog from *P. falciparum*

Structural alignment analysis using DALI (Holm & Rosenström, 2010) shows the high structural similarity of C1ORF123 with functionally unknown MAL13P1.257 protein from tropical pathogens *P. falciparum* (PDB ID: 1ZSO) that shares ∼26% of sequence identity with an RMSD of 1.9 Å (*Z*-score = 21). The protein also shares structural similarity with the CRY23AA1 protein (PDB ID: 4RHZ-B (*Z*-score = 3.5), Phospholipase A2 (PDB ID: 1RLW) (*Z*-score = 3.0) and O-glynacase NAGJ (PDB ID: 2JH2) (*Z*-score = 2.9) that have <10% sequence identity. Structure comparison of rC1ORF123 and MAL13P1.257 further shows that the biggest deviation is located in the zinc-binding region (Fig. 5C). It needs to be noted that MAL13P1.257 does not contain the CXXC metal-ion binding motifs. The results indicate that the human and *P. falciparum* homologues of DUF866 are highly similar at the C-terminal part of the molecule that consists of the beta strands β11–β12–β13–β14. On the other hand, the N-terminal variant composed of the beta strands β4–β5–β7–β8 is more variable (Fig. 5C).

### Identification of C1ORF123 interacting partner protein

Protein-protein interaction studies are powerful approach for functional characterization of protein with unknown function. Hence, we applied immnunoprecipitation (IP) to study C1ORF123 protein using rabbit polyclonal anti-C1ORF123 antibody. The anti-C1ORF123 antibody was first verified for reaction with rC1ORF123 by Western blot using anti-PentaHis-HRP (Qiagen, Valencia, CA, USA) (Fig. S2). A list of false positive proteins from HeLa cell lysate that unspecifically bound to the rabbit IgG was identified (Table S1). Our IP study has successfully identified four potential interacting partner proteins of C1ORF123 from human HeLa cell line (ATCC^^®^^ CCL-2^™^) lysate namely ATP synthase subunit alpha (ATP5A), dihydrolipoyllysine-residue succinyltransferase component of 2-oxoglutarate dehydrogenase (DLST), small ribosomal subunit proteins RPS13 and RPS15 (Table 2 and Fig. S3).

**Table 2:** The human C1ORF123 and its interacting protein partners identified by immunoprecipitation using rabbit anti-C1ORF123.

**No.**	**Protein name**	**UniProt ID**	**Molecular size(kDa)**	**Peptide**	**Peptide coverage(%)**
1.	UPF0587 protein C1orf123	Q9NWV4	18.0	4 [R].ENSIEILSSTIKPYNAEDNENFK.[T] [K].AQESVGIYEVTHQFVK.[C] [R].GLEPVDFQPQAGFAAEGVESGTAFSDINLQEK.[D] [K].TIVEFECR.[G]	49.3
2.	ATP synthase subunit alpha, mitochondria	P25705	59.7	5 [R].TGAIVDVPVGEELLGR.[V] [R].EVAAFAQFGSDLDAATQQLLSR.[G] [K].AVDSLVPIGR.[G] [K].TGTAEMSSILEER.[I] [K].TSIAIDTIINQK.[R]	13.2
3.	Dihydrolipoyllysine-residue succinyltransferase component of 2-oxoglutarate dehydrogenase complex, mitochondrial	P36957	48.7	5 [K].LGFMSAFVK.[A] [R].EAVTFLR.[K] [R].DYIDISVAVATPR.[G] [K].VEGGTPLFTLR.[K] [R].GLVVPVIR.[N]	10.5
4.	40S ribosomal protein S13 (Small ribosomal subunit protein uS15)	P62277	17.2	4 [K].GLTPSQIGVILR.[D] [K].SKGLAPDLPEDLYHLIK.[K] [K].GLAPDLPEDLYHLIK.[K] [K].KGLTPSQIGVILR.[D]	19.8
5.	40S ribosomal protein S15 (RIG protein) (Small ribosomal subunit protein uS19)	P62841	17.0	3 [R].GVDLDQLLDMSYEQLMQLYSAR.[Q] [R].KFTYRGVDLDQLLDMSYEQLMQLYSAR.[Q] [R].DMIILPEMVGSMVGVYNGK.[T]	31.7

### Phenotype characterization in *Schizosaccharomyces pombe*

To further decipher the function of C1ORF123, we have investigated its counterpart gene SPBC2D10.03c of *S. pombe* (hereby named as SpEss1 for S. *p**ombe* DUF **E**ight-**S**ix-**S**ix) that shared 54% of the sequence similarity. Both C1ORF123 and the 3D model of SpEss1 share closely similar structure with an RMSD of 0.35 Å (Fig. S4).

*S. pombe* sp*Ess1* knockout mutant strain (Δ*ess1*) was viable (Fig. S5A). However it exhibited slight temperature sensitivity at 36 °C (Figs. S5A, S5B), even though no significant cell morphology (Fig. S5C) or mitotic chromosome segregation phenotype was observed (Fig. S6) compared to the wild type. Consistent with lack of gross growth defects, Δ*ess1* also did not show any observable hypersensitivity towards the ribonucleotide reductase inhibitor hydroxyurea (HU) (Fig. S7A) and topoisomerase II inhibitor doxorubicin (DOXO) (Fig. S7B), which disrupts S-phase progression, and obstructs cell cycle events including chromosome segregation, respectively (Tay et al., 2013; Nguyen et al., 2015; Nguyen et al., 2016). All these observations suggest that SpEss1 is not directly involved in cell cycle control.

Intrigued by the possible functional connection of C1ORF123 to OXPHOS in mitochondria, we investigated the genetic interaction between the C1ORF123 counterpart gene of fission yeast, *ess1*^+^ with two genes encoding mitochondrial proteins, *coq10*^+^ and *tim11*^+^, on exposure to hydrogen peroxide (H_2_O_2_). Coq10 is a mitochondrial ubiquinone binding protein and its absence compromises respiration (Cui & Kawamukai, 2009), whereas *Tim11* encodes the ATP synthase subunit e (ATP21) of F1FO-ATPase. Knockout of *tim11* has been shown to reduce the structural dimerization and activity of F1F0-ATP synthase (OXPHOS complex V) in budding yeast (Arnold et al., 1998). H_2_O_2_ is a potent oxidizing agent that generates reactive oxygen species (ROS). Cells with disrupted mitochondrial OXPHOS are unable to sequester and transfer electrons through the OXPHOS complexes thereby exhibit hypersensitivity to this agent (Wong et al., 2017; Miki et al., 2008).

**Figure 6:**
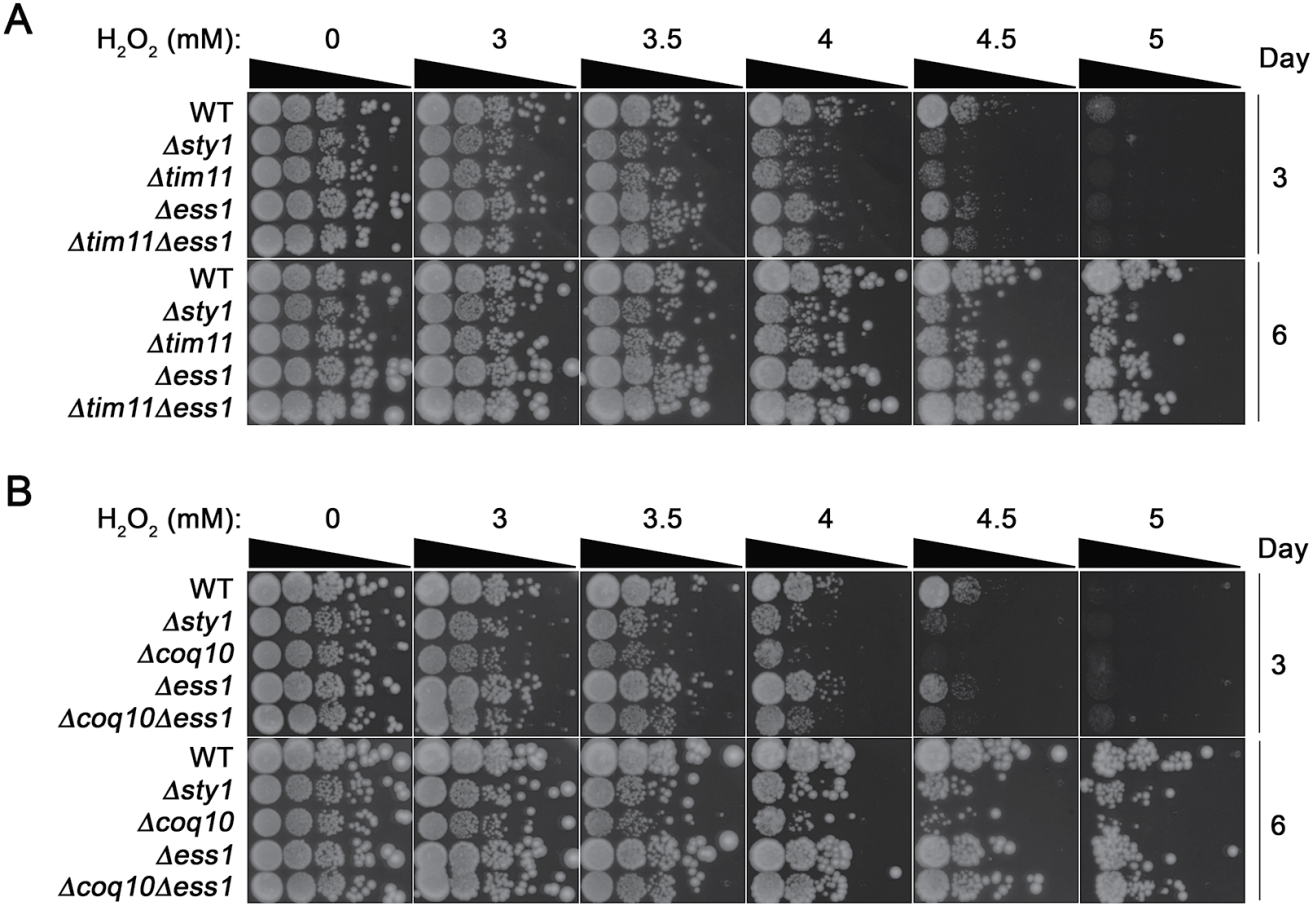
Positive genetic interaction between Ess1 and mitochondrial oxidative phosphorylation regulators in fission yeast. Hypersensitivity to hydrogen peroxide (H_2_O_2_) of null mutations of *ess1* and (A) *tim11* and (B) *coq10* were tested by exposing to the oxidizing agent. Cells were serially diluted and spotted onto YEA media containing 0, 3, 3.5, 4. 4.5 and 5 mM of H_2_O_2_. Cell growth was documented for 3 and 6 days after spotting. Δ*sty*1 strain was employed as a positive control. The result is a representative of three independent spotting experiments.

Consistent with such reported phenotype, null mutants of *tim11* and *coq10* (Δ*tim11* and Δ*coq10*) showed 10–100X more susceptibility to H_2_O_2_ compared to WT cells at 3.5–5 mM H_2_O_2_ (Figs. 6A, 6B). Δ*ess1* on the other hand did not exhibit reduced tolerance but grew as well as wildtype (WT) cells on media incorporated with H_2_O_2_ (Figs. 6A, 6B). However disruption of *ess1* in cells lacking these mitochondrial genes enhanced the tolerance of *tim11* and *coq10* towards H_2_O_2_ (Figs. 6A, 6B). Comparing to the single Δ*tim11* and Δ*coq10* mutants, the Δ*tim11* Δ*ess1* and Δ*coq10*Δ*ess1* double mutants showed approximately 100 folds (Fig. 6A) and 1,000 folds (Fig. 6B) more growth, which was particularly visible at 4.5 mM H_2_O_2_ (day 6). This suppressive effect was apparent already at the intermediate growth phase at day 3, but became highly prominent when the cells reached stationary phase at day 6 (Fig. 6). Such growth suppression, however, was not observed when the mutants were exposed to the cell cycle poison HU, suggesting the specificity towards the ROS generating agent (Fig. S7A). Although detailed mechanistic implication of this increase in H_2_O_2_ is currently unclear, these results suggest a link of Ess1 to mitochondrial respiratory system.

## Discussion

### Structure and functional analysis of C1ORF123 and DUF 866 family protein

The crystal structure of rC1ORF123 that consists of 160 amino acids has revealed an internal symmetry between N-terminal and C-terminal halves. Both halves that share low sequence identity were found to share significant structural fold, which was also observed in its *P. falciparum* counterpart MAL13P1.257. Structure and sequence analysis of both C1ORF123 and MAL13P1.257 shows that C-terminal half is more conserved than N-terminal across the two species. In human, there are two C1ORF123 transcript variants found to lack one or two alternation in-frame exons in the 5′ end are expected to produce proteins with truncated N-terminus or different structural conformation (Fig. S9). Both inter-species deviation and intra-species variations of the N-terminus that are observed in C1ORF123 further suggest that the C-terminal half is a more conserved domain than N-terminal half for DUF866 proteins. In human C1ORF123, four post-translational modification (PTM) residue sites involving phosphorylation (Y45, S51, and Y151) and ubiquitination (K55) have been identified (Lee et al., 2006). Three of these residues are located at the C1ORF123 N-terminal region, in particular, S51 and K55 are located at the η1, β5 and β6 insertion of the N-terminal half, suggesting that this insertion may play functional role that yet to be known of the protein.

**Figure 7:**
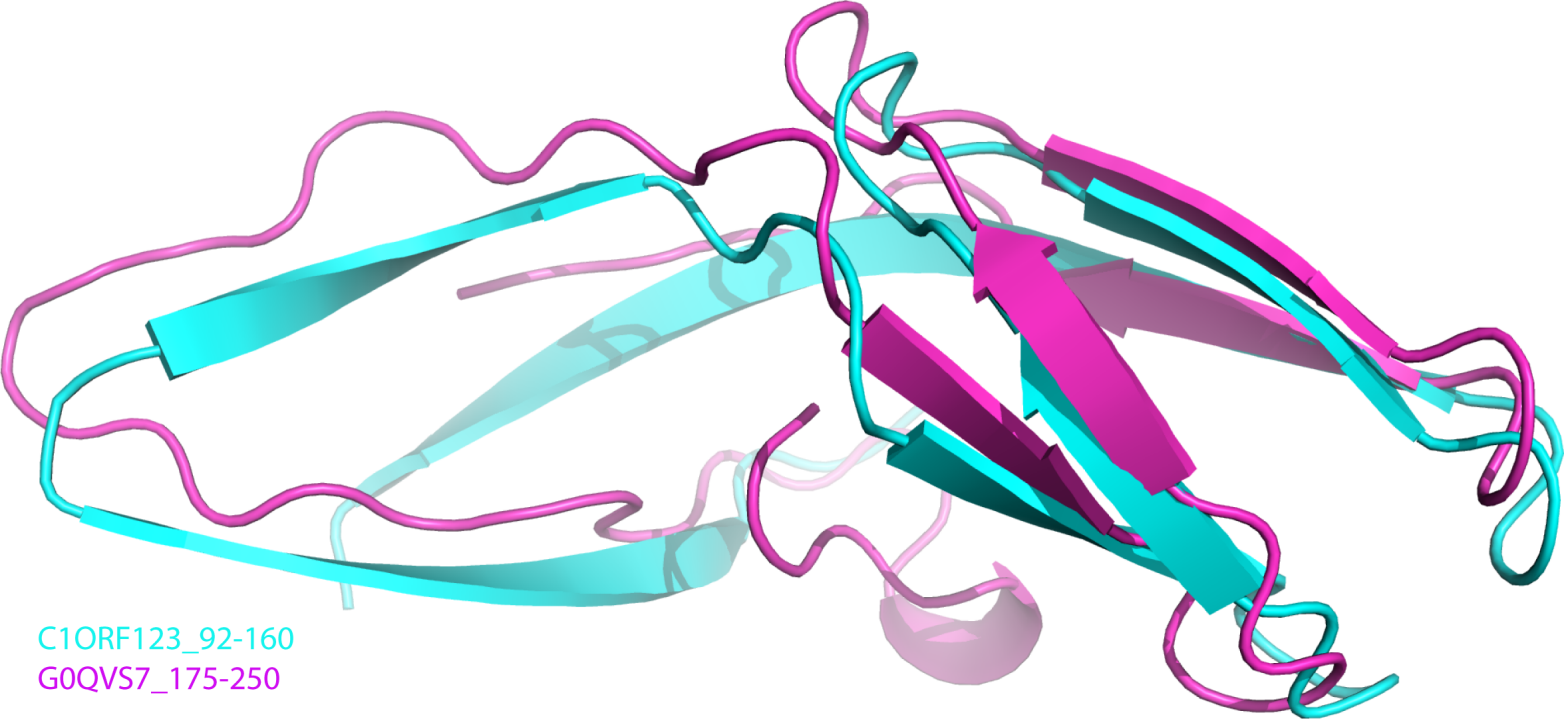
Superposition of C1ORF123 C-terminal half and DUF 866 domain of protein G0QVS7. The human C1ORF123 C-terminal half, residue 92–160 (cyan) and 3D structure was superimposed with DUF866 domain of carbon-nitrogen family protein (residue 175–250), G0QVS7 of *Ichthyophthirius multifiliis* (magenta) that predicted using Phyre2.

To further analyze the N-terminal and the C-terminal halves of DUF866 protein family, a DUF866 domain that consists of 75 residues found in a carbon-nitrogen family protein, namely G0QVS7 of *Ichthyophthirius multifiliis*, an ectoparasite protozoan that causes freshwater white spot disease in fish was investigated. The G0QVS7 protein contains 257 amino acids and consists of a DUF866 domain (residues 175–250) and a CN hydrolase domain that is important for hydrolysis of non-peptide carbon-nitrogen bonds. The 75 amino acids DUF866 domain (DUF866_175-250) was used for the 3D structure prediction using Phyre2 (Kelley et al., 2015). The predicted structure of DUF866_175-250 domain from G0QVS7 was superposed onto C1ORF123. The fold of G0QVS7 DUF866_175-250 was indeed very similar to the C-terminal half of C1ORF123 with an RMSD of 1.5 Å (63 Cα atoms of aligned residues) and 25% sequence identity (Fig. 7). This observation also suggests that half of the C1ORF123 protein could stand alone as an individual domain. In combination with the internal symmetry observed in C1ORF123, we suggest that the DUF866 protein family may have evolved through internal domain duplication.

Sequence analysis also revealed that most (∼90%) of the DUF866 proteins contain a CX _2_CX _30_CX _2_C motifs (Fig. S1) at the N-terminal half. Same motif is also present in the E7 proteins of Papillomavirus (García-Pérez et al., 2014). A similar sequence motif is also found in the iron or zinc binding motif CX _2_CX _29_CX _2_C of the Rub protein from *Clostridium thermoaceticum*, the human papillomavirus E6, the E6 and E7 protein of *Epidermodysplasia verruciformis* (EV) (Das et al., 2001; Tobler et al., 2006; Sobhy, 2016). Protein-ligand binding site prediction using COACH program (Yang, Roy & Zhang, 2013) has identified a similar zinc-binding motif (*C*-score = 0.31) at the C-terminal domain of human RIG-I-like receptor LGP2 (PDB ID: 3EQT) (Fig. 2E), which interacts with viral RNA as a part of the innate imMune response (Li et al., 2009). A similar motif was also identified in the regulatory domains of RIG-I (retinoic acid inducible gene 1 protein, PDB ID:2QFB) and MSS4 (GDP/GTP exchange factor for small Rab-like GTPases) (PDB ID: 2FU5) (Yu & Schreiber, 1995; Itzen et al., 2006; Cui et al., 2008) (Fig. 2E). Mutations of these cysteine residues in the regulatory domain of RIG-I have shown that the zinc-binding is essential for the *in vivo* protein function which involves RIG-I regulation (Cui et al., 2008). The zinc-binding site in MSS4 was found to interact with Rab8 GTPase in the crystal structure of MSS4-Rab8 complex, suggesting its role in modulating the nucleotide-binding site of the GTPase (Itzen et al., 2006). Given that the *P. falciparum* homologue of C1ORF123 was found as a stable protein despite lacking the CX _2_CX _30_CX _2_C motifs that bound zinc ion (Holmes et al., 2006), it is likely that the main function of the zinc-binding motif of C1ORF123 is not exclusively for structural stability.

In agreement to this, a cavity that located next to the zinc-binding motif was identified in molecules A and B of C1ORF123 to assemble open- and closed-form conformation, respectively. The open-close conformation of the cavity is mediated by two ‘gate mediating’ residues D41 and K68. The glycerol molecule in the closed-form cavity interacts via hydrogen bonds with the main chain O atom of residues M31 and E38, main chain N atom of K68, and a water molecule that hydrogen bonded with the side-chain O atom of N74. The coordination of the water molecule is conserved in both the molecules A and B. A smaller cavity size in the molecule B compared to the molecule A may be a result of the interaction of glycerol with the surrounding residues. This interaction subsequently closes the pocket and causes the deviation of the loop β7–β8 by 1.4 Å (Fig. 3C), which presumably represents the closed form of the cavity. Note that a similar cavity does not exist in the *P. falciparum* homologous structure due to presence of a bulky tryptophan side chain, which occupies the glycerol-binding pocket (Fig. 3D). Many DUF866 proteins that do not have the zinc-binding motif were also found to have the bulky side chain residue (tryptophan, phenylalanine and histidine) (Fig. S1). The tryptophan residue is equivalent to one of the Cys residue (C67) for CX _2_CX _30_**C**X _2_C motif in C1ORF123. This observation suggests that the glycerol-binding cavity may result from the zinc-binding domain formation. Both zinc-binding site and glycerol binding cavity found in C1ORF123 is likely to play functional roles that are yet to be discovered. Their potential function may involve protein regulation or nucleic acid interaction similar to the zinc binding site of RIG-1, RIG-I-like receptor LGP2 and MSS4 (Yu & Schreiber, 1995; Itzen et al., 2006; Cui et al., 2008; Li et al., 2009).

### DUF866 protein functionally related to oxidative phosphorylation system

To further understand the function of C1ORF123 protein, the IP study has been conducted using rabbit polyclonal anti-C1ORF123 antibody. Four protein partners have been identified including two mitochondrial proteins, ATP5A and DLST. DLST is a component protein of 2-oxoglutarate dehydrogenase complex that catalyzes the conversion of 2-oxoglutarate to succinyl-CoA and carbon dioxide in mitochondria, while ATP5A is encoded for a catalytic core subunit of domain 1 in mitochondrial ATP synthase that is important for ATP synthesis (Collinson et al., 1996; Houštěk, Kmoch & Zeman, 2009). Disruption of ATP5A in ATP synthase has been shown to cause mitochondrial reactive oxygen species generation (Ni et al., 2015). Moreover, inhibition of the ATP5A O-GlcNAcylation has been reported to play role in neurodegenerative diseases such as Alzheimer’s disease (Cha et al., 2015). As C1ORF123 was previously identified as an O-GlcNAc transferasae (OGT) interactor (Deng et al., 2014) and in combination with previous proteomics data, it showes that C1ORF123 may also be involved in psychotic diseases and age-related changes in brain function (Schubert, Föcking & Cotter, 2015; Wearne et al., 2014; Vazquez, Hall & Greco, 2009). These findings led us to hypothesize that C1ORF123 protein may interact with those mitochondrial proteins identified in our IP experiment to functionally regulate oxidative phosphorylation system (OXPHOS). OXPHOS is known to be important for neuronal development and plasticity, and synapse connectivity (Bergman & Ben-Shachar, 2016). The immunoprecipitation experiment also identified two small ribosomal subunit proteins (RPS13 and RPS15) as C1ORF123-interacting factors. However, due to a number of ribosomal proteins that were also detected upon immunoprecipitation with control rabbit IgG antibody (Table S1), we cannot rule out the potential of false positives.

The suppression of H_2_O_2_ hypersensitivity associated with the OXPHOS-defective Δ*tim11* and Δ*coq10* mutants by concurrent ablation of *ess1* concurs with the proposed OXPHOS-related role for human C1ORF123. Tim11 is an ATP synthase complex subunit that coordinates the dimerisation and oligomerization of the complex, which in turn regulates the morphogenesis of the inner mitochondrial membrane to generate the mitochondrial tubular cristae (Rabl et al., 2009; Wagner et al., 2009). Increased fragmentation of mitochondria is expected to occur in the absence of Tim11 function that underlies respiratory defect in the cells to predispose them to be H_2_O_2_ hypersensitive (Amutha et al., 2004). Mitochrondrial fragmentation however is an essential event during cell division when the tubular cristae disassembles for organellar distribution into the progeny cells. Although the molecular mechanism is currently elusive, this process can involve temporal downregulation of Tim11 function to counteract F_1_F_0_ATP synthase multimerization (Arnold et al., 1998; Everard-Gigot et al., 2005). It is possible for Ess1 to negatively regulate and keep the mitochondrial fragmentation in check until the appropriate time. Consequently, loss of morphological integrity of the mitochondrial inner membrane associated with Δ*tim11* can conceptually be side-stepped by the loss of *ess1*. Consistent with this view, regulators of membrane fusion and cell cycle were reported to interact with human C1ORF123 as noted in our previous work (Rahaman et al., 2016).

OXPHOS can be coordinated through signaling pathways in response to environment nutritional status in *S. pombe* (Shah et al., 2016). Alternatively, Ess1 may act to relay such signal to repress OXPHOS, envisaged to be essential when inappropriate external cues are detected. Loss-of-function of *tim11* and *coq10* compromises electron transfer efficiency between the respiratory complexes (Arnold et al., 1998; Amutha et al., 2004), which may contribute to the H_2_O_2_ hypersensitivity. Hence, deletion of *ess1* in *tim11* and *coq10* backgrounds can lift the repression on OXPHOS to boost the capability of Δ*tim11* and Δ*coq10* to handle the deleterious effect of the oxidizing agent as observed in Fig. 7. These two possible impacts of Ess1 on OXPHOS are not mutually exclusive. However, in view of the similar effect of Δ*ess1* on both Δ*tim11* and Δ*coq10,* the influence on OXPHOS would be more plausible, which will await further confirmation in future work.

Together with the interaction of C1ORF123 with ATP5A and DLST, our results contributed to explanation of a physiological link between DUF866 proteins with mitochondria-related processes, probably the oxidative phosphorylation and energy metabolism of the cell. This hypothesis will facilitate future research to address the details of the mechanisms of action in the context of regulation of energy metabolism of the cell.

The N-terminal half of C1ORF123 that contains a zinc ion bound by the zinc-finger motif, likely represents the key reaction centre domain as it is conserved across almost all of the DUF866 family members, especially those that contain standalone DUF866 domain, including the 157 residues SpEss1. Furthermore, C1ORF123 residues D41 and K68 that mediate the open-close confirmation of the observed glycerol binding cavity are also conserved in spEss1 (corresponding residues D42 and K69) (Fig. S4A). Based on these findings, we propose for the future studies to focus on the structural and functional elucidation of the conserved zinc binding motif and the role of potential functional residues of the DUF866 family in OXPHOS regulation.

## Conclusion

The structure of human C1ORF123 protein has been successfully determined to atomic resolution. Structure analysis of C1ORF123 suggesting that it has probably underwent an internal domain duplication event to produce N-terminal and C-terminal domains that differ in electrostatic potential surfaces and have presumably diverged for different functions. Furthermore, the crystal structure revealed the zinc binding motif at the N-terminal domain that is conserved for majority of the DUF866 protein family members, but is absent in the *Plasmodium falciparum* homologuous structure. A cavity that underwent conformational changes upon binding of a glycerol molecule was revealed in this crystal structure, suggesting that C1ORF123 may interact with small molecule despite its function is yet to be determined. Functional studies of C1ORF123 and its counterpart in *S. pombe* suggest a role of DUF866 proteins in oxidative phosphorylation in mitochondria. With the high-resolution structure of C1ORF123 being now available, structure guided mutagenesis can be applied for future functional studies of the C1ORF123 and DUF866 family proteins.

##  Supplemental Information

10.7717/peerj.5377/supp-1 Supplemental Information 1 Sequence alignment of selected 499 protein sequences of DUF866 family that contain DUF866 stand alone domain within 150–170 amino acids using CLUSTALX (Larkin et al., 2007) Conserved regions of two CXXC motifs are shown in pink highlights, while proteins without CXXC motifs that are exclusively found in apicomplexans, oomycetes, algae, choanoflagellate and phytoplankton species are highlighted in blue boxes.

10.7717/peerj.5377/supp-2 Supplemental Information 2 Sequence alignment of selected 499 protein sequences of DUF866 family that contain DUF866 stand alone domain within 150–170 amino acids using CLUSTALX (Larkin et al., 2007) Conserved regions of two CXXC motifs are shown in pink highlights, while proteins without CXXC motifs that are exclusively found in apicomplexans, oomycetes, algae, choanoflagellate and phytoplankton species are highlighted in blue boxes.

10.7717/peerj.5377/supp-3 Supplemental Information 3 Immunoprecipitation of rC1ORF123 with anti-C1ORF123 antibody varified by Western blot using anti-PentaHis-HRP (Qiagene, USA) (A) SDS-PAGE (12.5%) electrophoresis analysis, M: protein marker (Thermo Scientific PageRuler Plus Prestained Protein Ladder); Lane 1: rC1ORF123; Lane 2: anti-C1ORF123 antibody; Lane 3: HeLa cell lysate; Lane 4: Elution of anti-C1ORF123 immunoprecipitated rC1ORF123 (B) Western blot analysis using anti-PentaHis-HRP (Qiagen, USA) for (A).

10.7717/peerj.5377/supp-4 Supplemental Information 4 Potential interacting partners of C1ORF123 identified using Immunoprecipitation Venn diagram shows identified proteins from Immunoprecipitation experiment of control (rabbit IgG polyclonal + HeLa cells lysate), sample antiC1_HeLa (rabbit anti-C1ORF123 + HeLa cells lysate) and antiC1_rC1_HeLa (rabbit anti-C1ORF123 + rC1ORF123 proein + HeLa cells lysate). The C1ORF123 protein and its 4 potential interacting partners that only identified in both antiC1_HeLa and antiC1_rC1_HeLa are circled in red.

10.7717/peerj.5377/supp-5 Supplemental Information 5 Sequence alignment and structure comparison of C1ORF123 and SpEss1 (A) Sequence alignment of C1ORF123 and SpEss1 shows both proteins share high sequence similarity. The conserved *CX*_2_*CX*_30_*CX*_2_*C* motif was labeled in pink while the residues of Aspartate-41 (Aspartate-42 for SpEss1) and Lysine-69 (Lysine-70 for SpEss1) were shown with asterisk in green. (B) Superimposition of C1ORF123 (Green) and *S. pombe* homologue spEss1 (Cyan) obtained from Swiss-Model with C1ORF123 structure as template.

10.7717/peerj.5377/supp-6 Supplemental Information 6 Cell growth phenotypes of Δ*ess*1 fission yeast mutant (A) WT and Δ*ess*1 were streaked on YEA agar media and incubated at 20, 26, 30, 33 and 36 °C. Δ*ess*1 exhibited weak temperature sensitivity at 36° C. Growth was documented at 3 and 6 days. (B) WT and Δ*ess*1 cells were growth to log-phase at 30 °C, and cell growth was quantified by the measurement of optical density at 600 nm over 8 h. The result represents mean of two independent experiments. (C) Cell morphology of WT and Δ*ess*1 cells at 30 and 36 °C (8 h) bar: 3 µm.

10.7717/peerj.5377/supp-7 Supplemental Information 7 Δ*ess*1 did not show abnormal cell cycle phenotypes 300 post-mitotic cells with two or more nuclei were scored for the indicated phenotypes from Δ*ess*1 (grey) and WT (orange) cultures. The microscopic phenotype of the strains were inserted at the top right hand corner. Bar: 3 µm. N >300. All the categories were not significantly different from each other between the WT and Δ*ess*1 stains (*p* > 0.05) using two-tailed Student’s *t-* test.

10.7717/peerj.5377/supp-8 Supplemental Information 8 The *Schizosaccharomyces pombe* spEss1 knockout mutant shown no significant susceptibility to (A) hydroxyurea (HU) and (B) doxorubicin (DOXO) Δ*cds*1 and Δ*rav*1 are null mutants of the Cds1 replication checkpoint effector kinase and assembly factor of vacuolar-ATPase, which were employed as positive controls to show hypersensitivity to HU and DOXO respectively as in previous work (Nguyen et al., 2016).

10.7717/peerj.5377/supp-9 Supplemental Information 9 Δ*ess*1 did not show hypersensitivity towards hydroxyurea (HU) Log-phase WT and Δ*ess*1 cultures were ten-fold serially diluted and spotted on YEA media incorporated with the indicated concentrations of HU. Δ*cds*1 is the deletion mutant of the replication checkpoint kinase Cds1 and is used as a positive control as previously reported (Nguyen et al., 2016). Triangle: Serial dilution from more to less cell number. Plates were grown at 30 °C and documented after 7 days of growth.

10.7717/peerj.5377/supp-10 Supplemental Information 10 Sequence alignment and structure comparison of C1ORF123 and its two transcript variants (A) Sequence alignment of C1ORF123 and its two transcript variants that lack of one (isoform 2) and two (isoform 3) alternate in-frame exon in the 5’ end. (B) The 3D model structure of isoform 2 and 3 obtained using I-Tasser (Zhang, 2008) shown to have more simplified structure compared to C1ORF123.

10.7717/peerj.5377/supp-11 Supplemental Information 11 The list of rabbit IgG co-immunoprecipitated human proteins from HeLa cells lysate. These proteins were identified as false positive for the immunoprecipitation experiment that conducted in this study

## Additional Information and Declarations

### Data Availability

The following information was supplied regarding data availability: The structure factor and coordinate files have been deposited to Protein Data Bank (PDB ID: 5ZRT).
