# Supplementary figures and images for: Crystal structure and functional analysis of human C1ORF123
Source: PeerJ. 2018 Sep 28;6:e5377. doi: 10.7717/peerj.5377 (PMC6166629; doi:10.7717/peerj.5377)

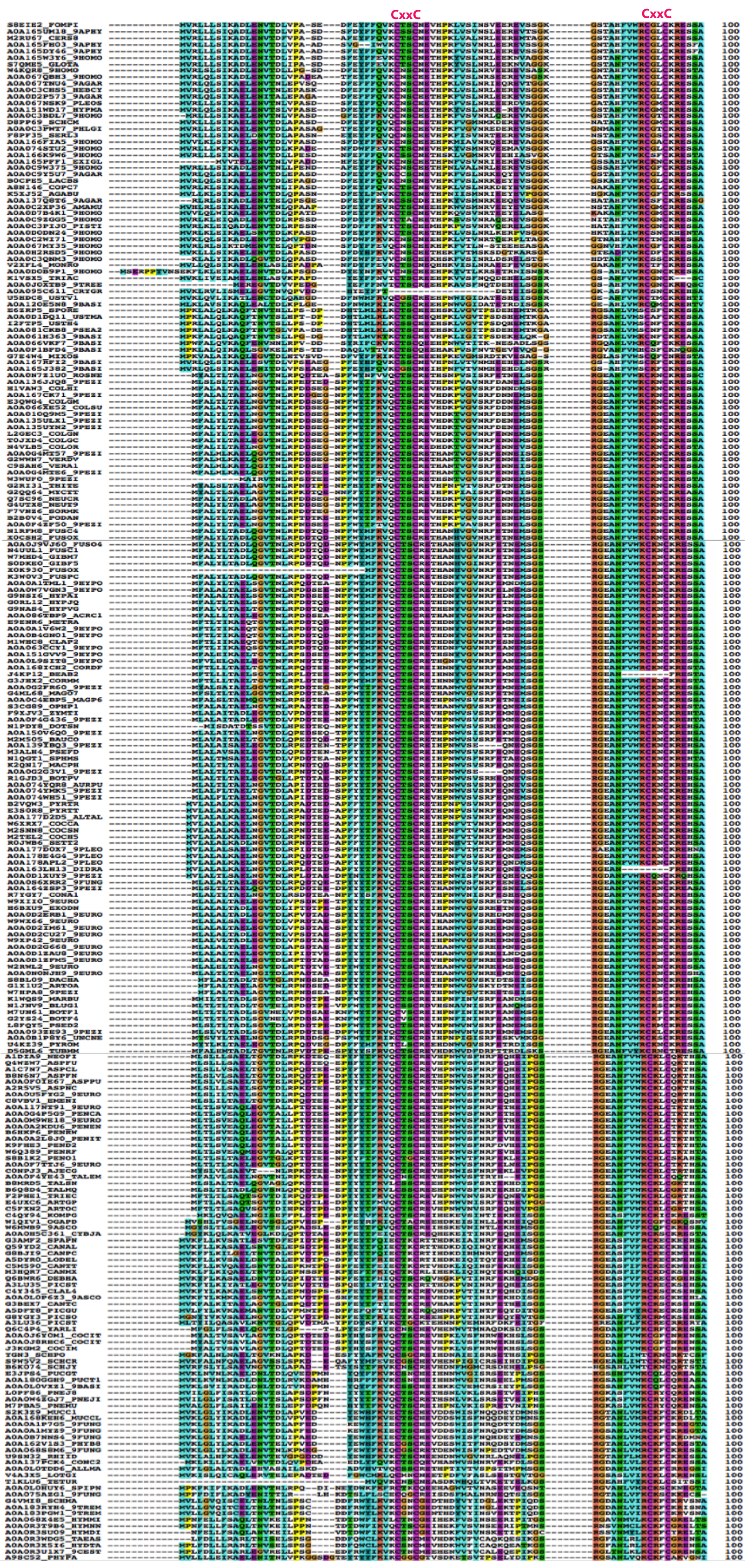

Supplement: Supplemental Information 1 — Conserved regions of two CXXC motifs are shown in pink highlights, while proteins without CXXC motifs that are exclusively found in apicomplexans, oomycetes, algae, choanoflagellate and phytoplankton species are highlighted in blue boxes. [file peerj-06-5377-s001.png]

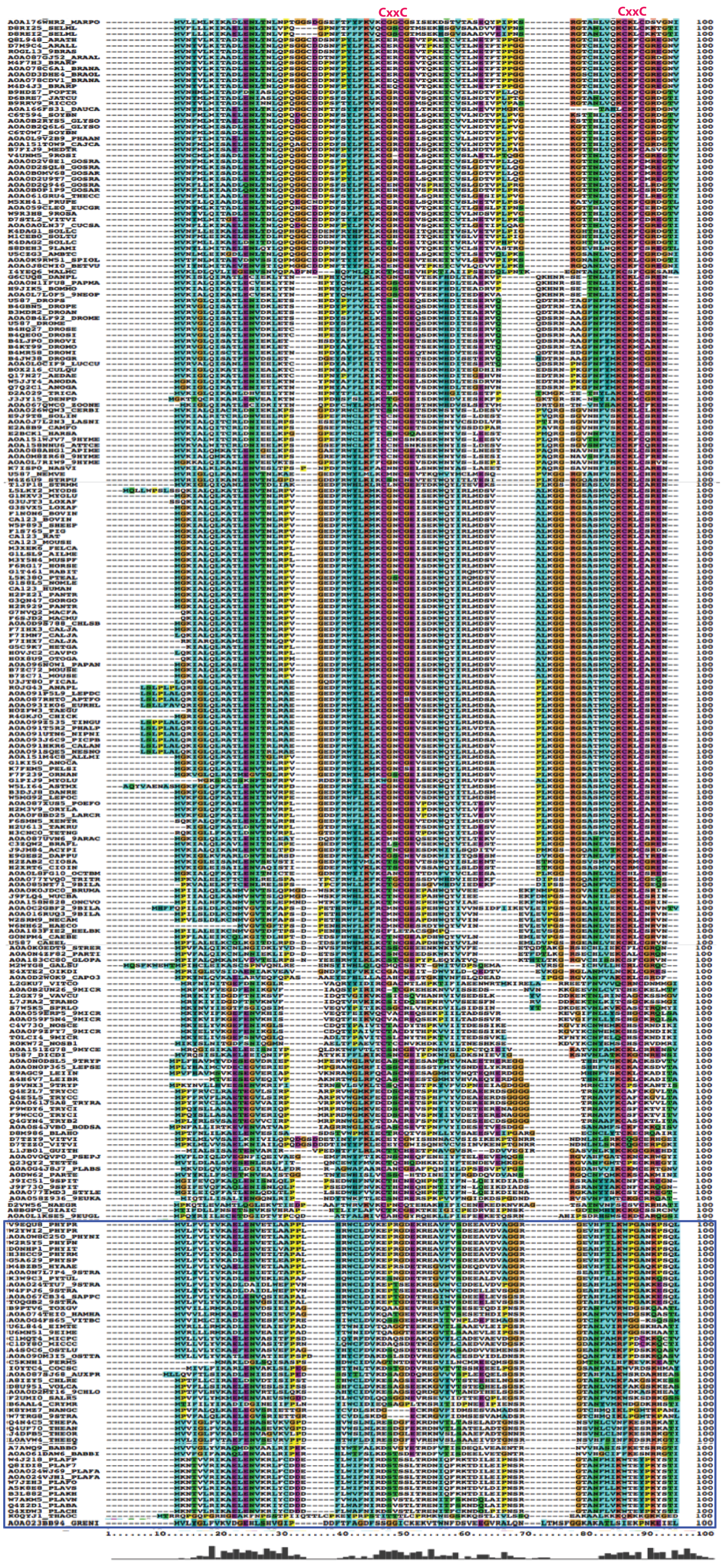

Supplement: Supplemental Information 2 — Conserved regions of two CXXC motifs are shown in pink highlights, while proteins without CXXC motifs that are exclusively found in apicomplexans, oomycetes, algae, choanoflagellate and phytoplankton species are highlighted in blue boxes. [file peerj-06-5377-s002.png]

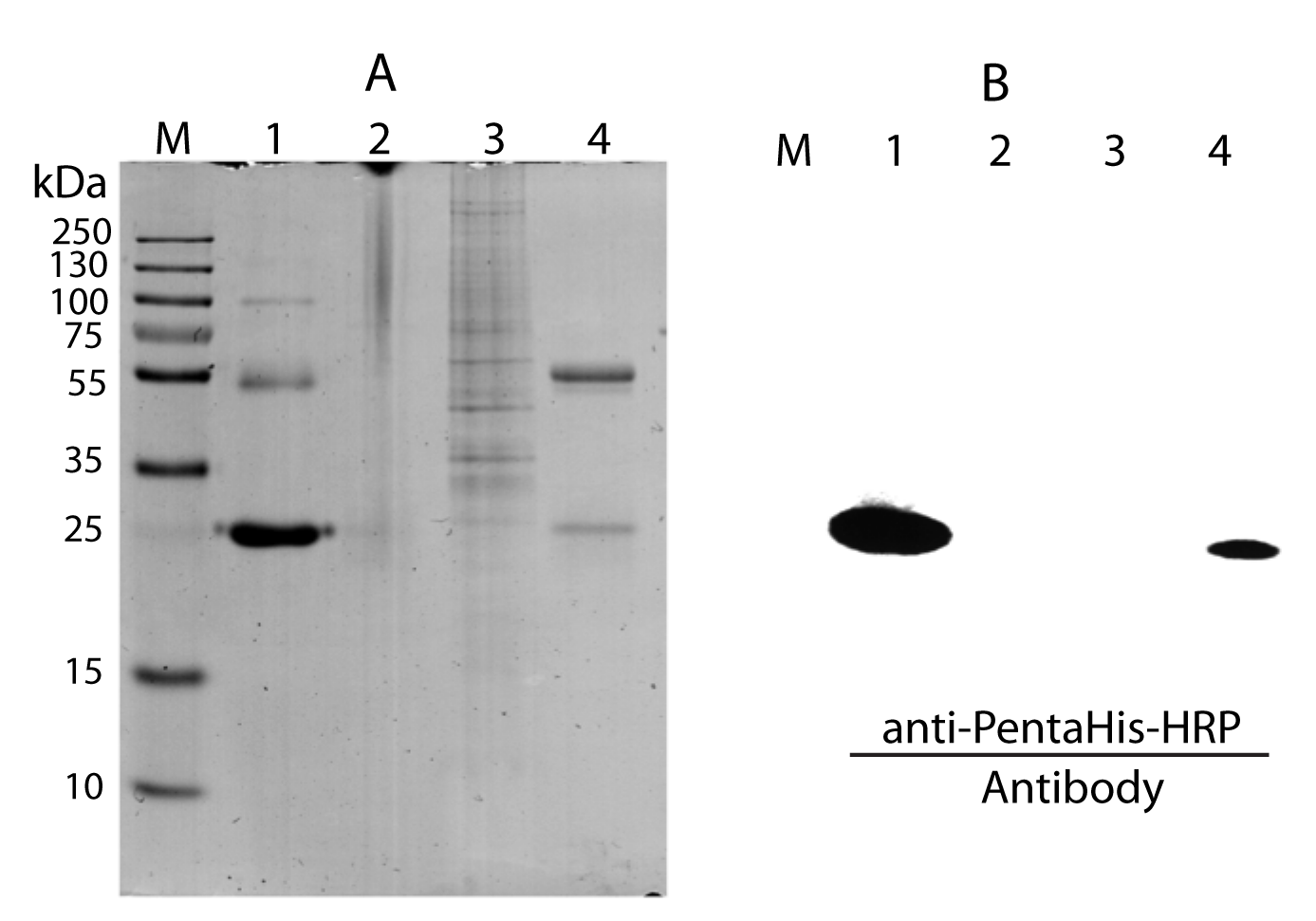

Supplement: Supplemental Information 3 — (A) SDS-PAGE (12.5%) electrophoresis analysis, M: protein marker (Thermo Scientific PageRuler Plus Prestained Protein Ladder); Lane 1: rC1ORF123; Lane 2: anti-C1ORF123 antibody; Lane 3: HeLa cell lysate; Lane 4: Elution of anti-C1ORF123 immunoprecipitated rC1ORF123 (B) Western blot analysis using anti-PentaHis-HRP (Qiagen, USA) for (A). [file peerj-06-5377-s003.png]

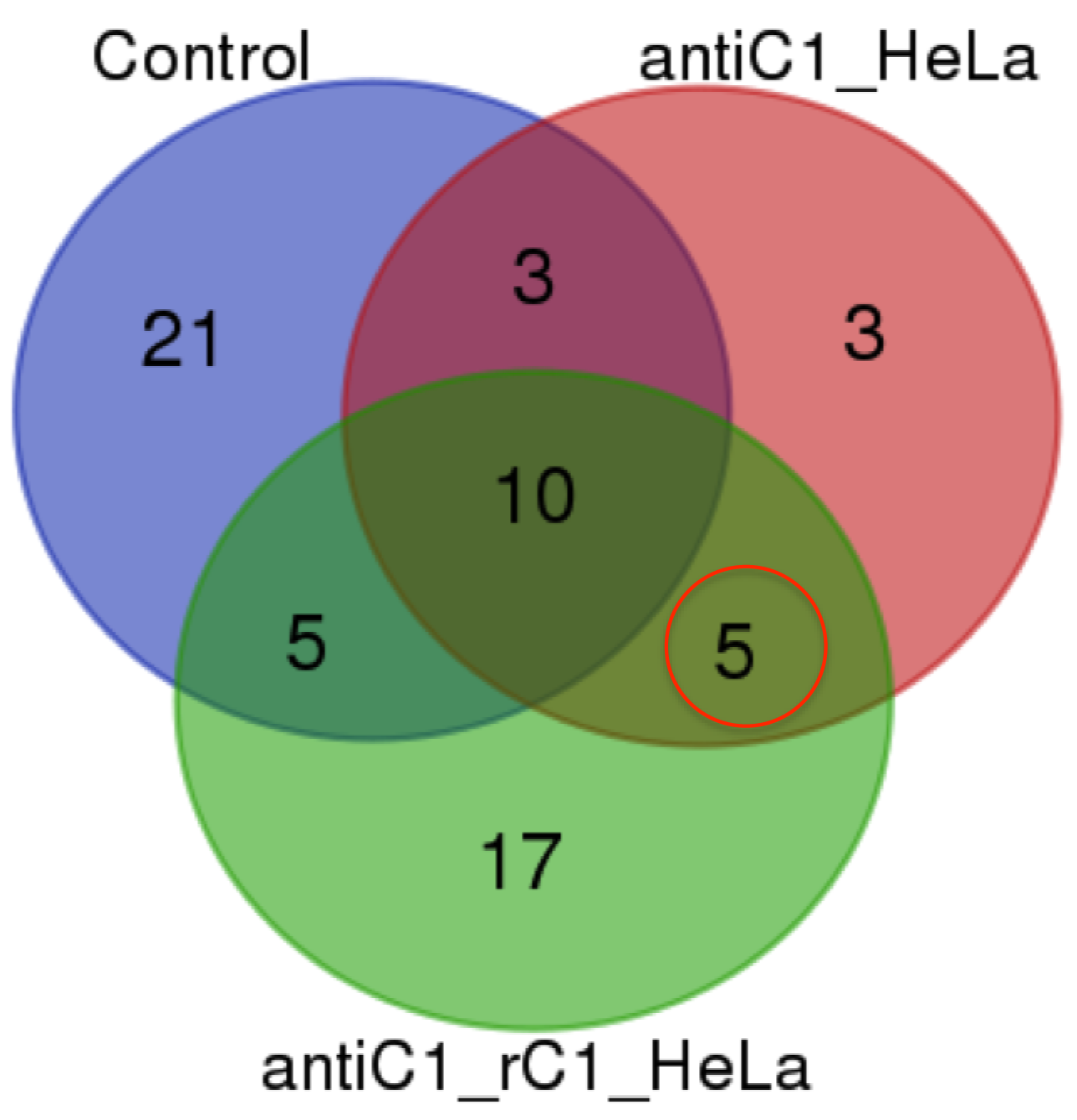

Supplement: Supplemental Information 4 — Venn diagram shows identified proteins from Immunoprecipitation experiment of control (rabbit IgG polyclonal + HeLa cells lysate), sample antiC1_HeLa (rabbit anti-C1ORF123 + HeLa cells lysate) and antiC1_rC1_HeLa (rabbit anti-C1ORF123 + rC1ORF123 proein + HeLa cells lysate). The C1ORF123 protein and its 4 potential interacting partners that only identified in both antiC1_HeLa and antiC1_rC1_HeLa are circled in red. [file peerj-06-5377-s004.png]

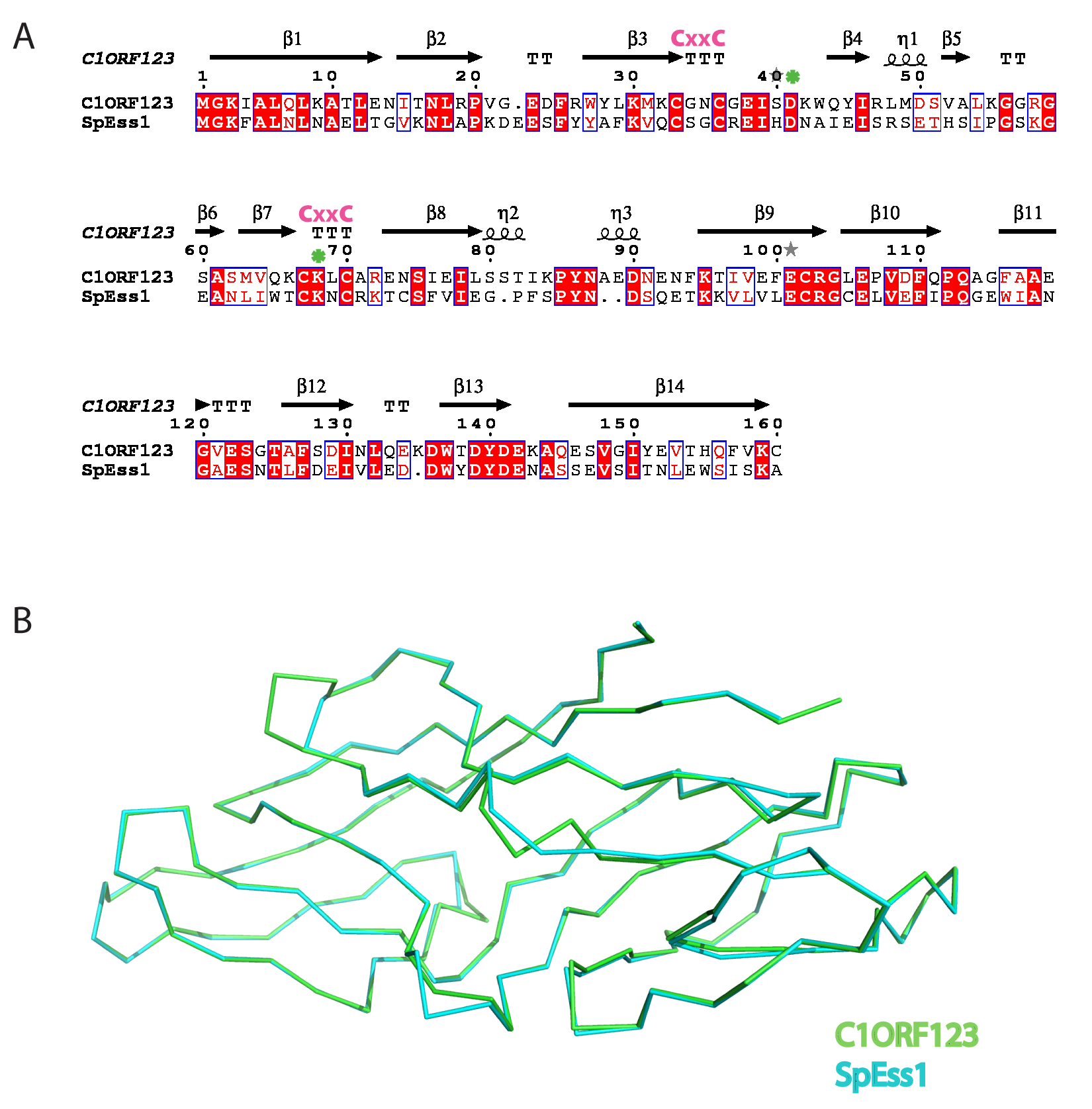

Supplement: Supplemental Information 5 — (A) Sequence alignment of C1ORF123 and SpEss1 shows both proteins share high sequence similarity. The conserved CX2CX30CX2C motif was labeled in pink while the residues of Aspartate-41 (Aspartate-42 for SpEss1) and Lysine-69 (Lysine-70 for SpEss1) were shown with asterisk in green. (B) Superimposition of C1ORF123 (Green) and S. pombe homologue spEss1 (Cyan) obtained from Swiss-Model with C1ORF123 structure as template. [file peerj-06-5377-s005.png]

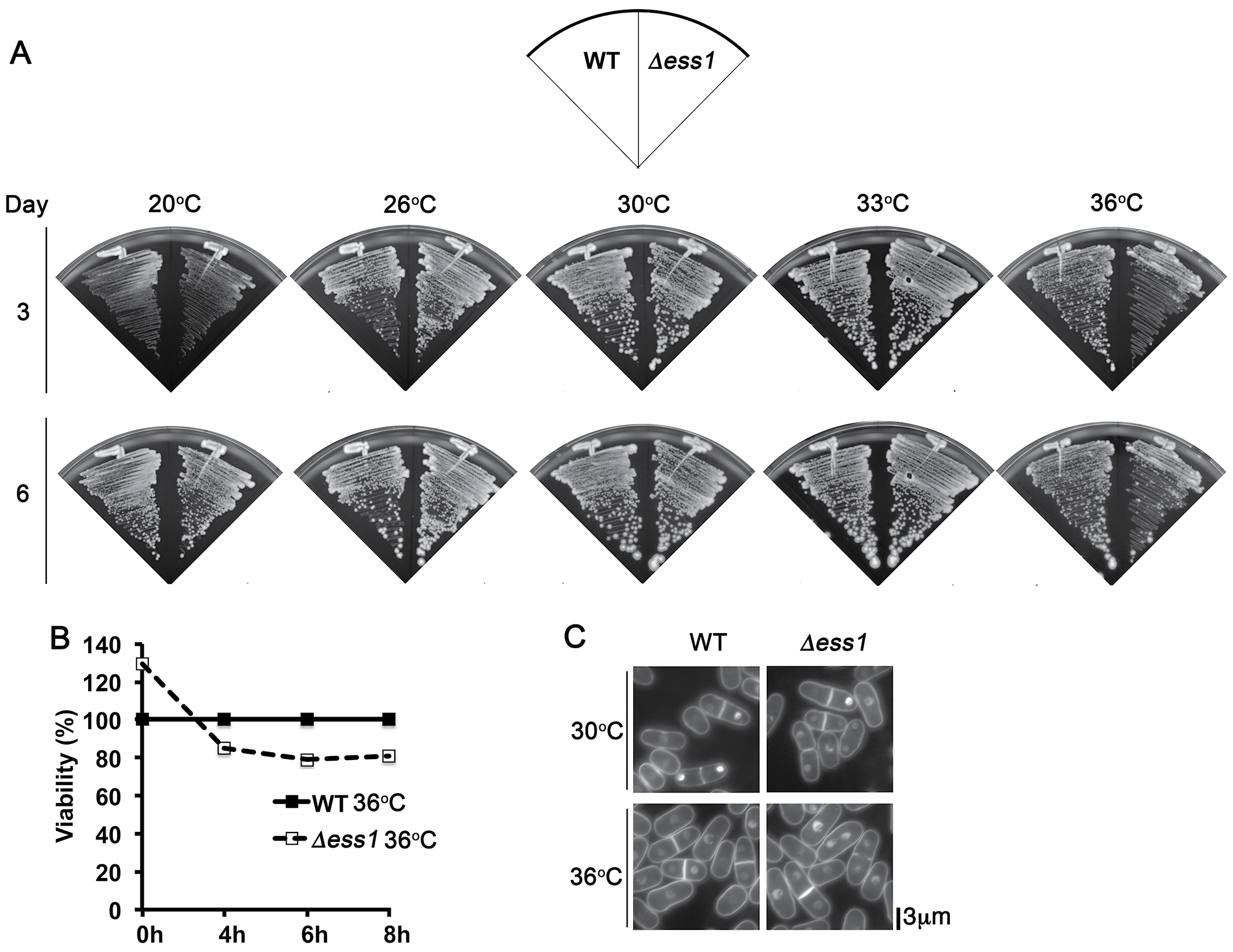

Supplement: Supplemental Information 6 — (A) WT and Δess1 were streaked on YEA agar media and incubated at 20, 26, 30, 33 and 36 °C. Δess1 exhibited weak temperature sensitivity at 36° C. Growth was documented at 3 and 6 days. (B) WT and Δess1 cells were growth to log-phase at 30 °C, and cell growth was quantified by the measurement of optical density at 600 nm over 8 h. The result represents mean of two independent experiments. (C) Cell morphology of WT and Δess1 cells at 30 and 36 °C (8 h) bar: 3 µm. [file peerj-06-5377-s006.png]

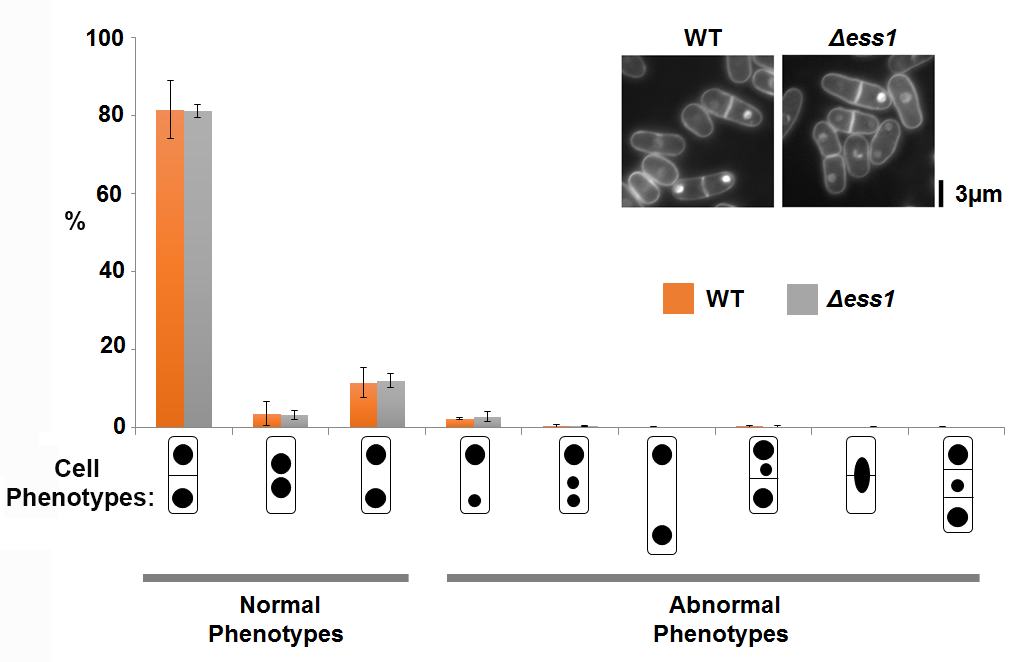

Supplement: Supplemental Information 7 — 300 post-mitotic cells with two or more nuclei were scored for the indicated phenotypes from Δess1 (grey) and WT (orange) cultures. The microscopic phenotype of the strains were inserted at the top right hand corner. Bar: 3 µm. N >300. All the categories were not significantly different from each other between the WT and Δess1 stains (p > 0.05) using two-tailed Student’s t- test. [file peerj-06-5377-s007.png]

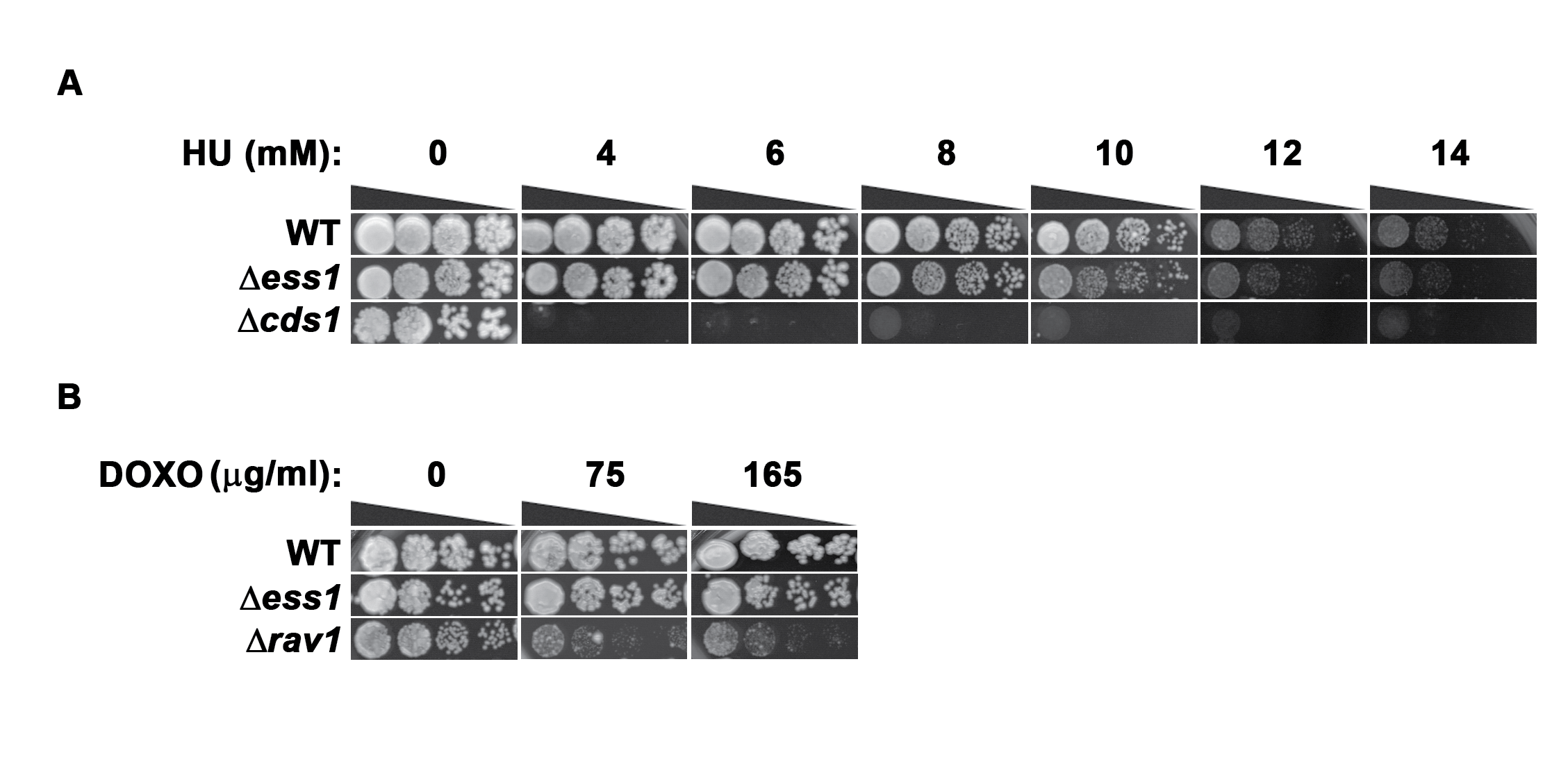

Supplement: Supplemental Information 8 — Δcds1 and Δrav1 are null mutants of the Cds1 replication checkpoint effector kinase and assembly factor of vacuolar-ATPase, which were employed as positive controls to show hypersensitivity to HU and DOXO respectively as in previous work (Nguyen et al., 2016). [file peerj-06-5377-s008.png]

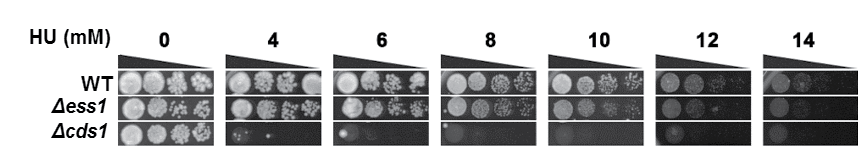

Supplement: Supplemental Information 9 — Log-phase WT and Δess1 cultures were ten-fold serially diluted and spotted on YEA media incorporated with the indicated concentrations of HU. Δcds1 is the deletion mutant of the replication checkpoint kinase Cds1 and is used as a positive control as previously reported (Nguyen et al., 2016). Triangle: Serial dilution from more to less cell number. Plates were grown at 30 °C and documented after 7 days of growth. [file peerj-06-5377-s009.png]

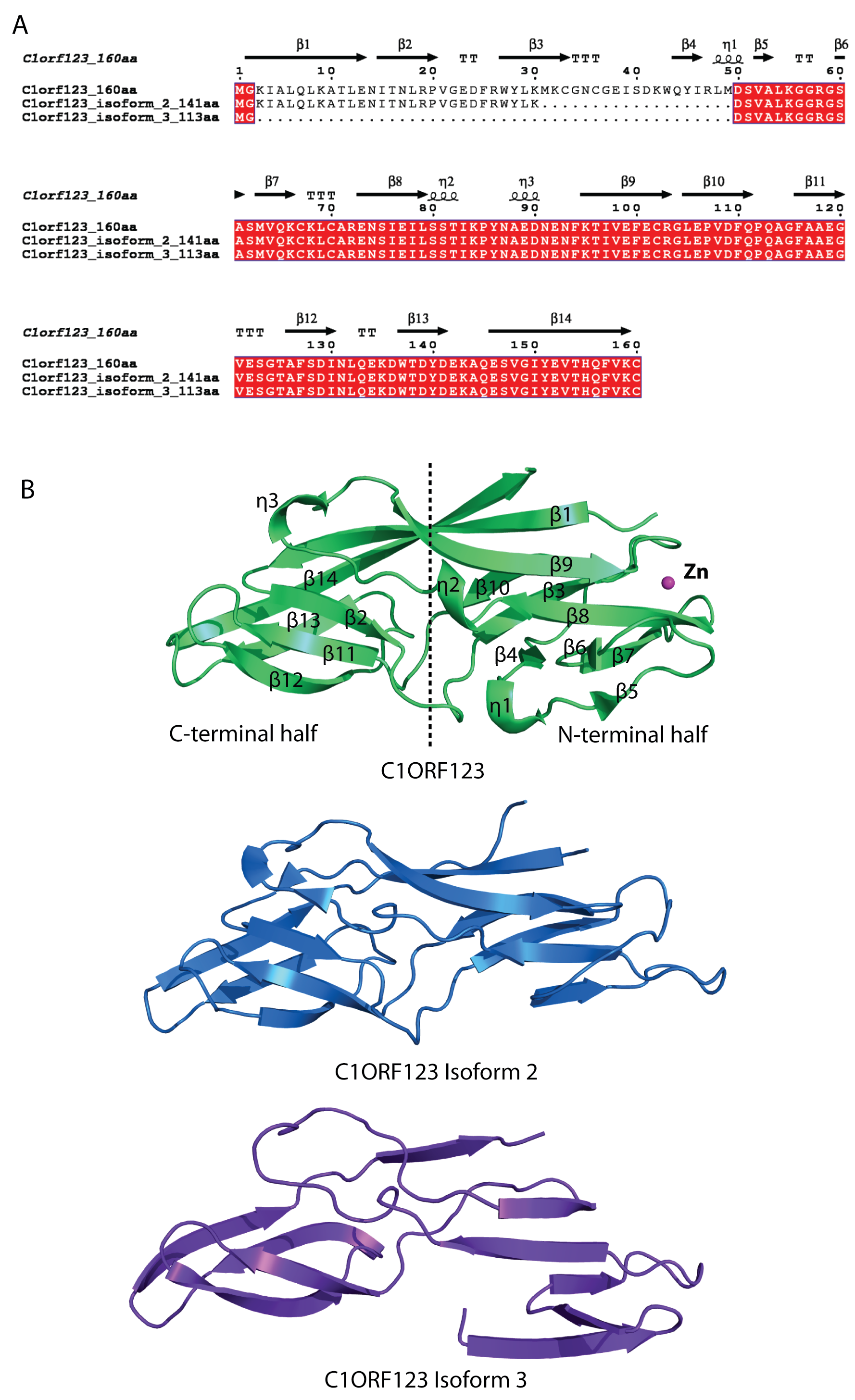

Supplement: Supplemental Information 10 — (A) Sequence alignment of C1ORF123 and its two transcript variants that lack of one (isoform 2) and two (isoform 3) alternate in-frame exon in the 5’ end. (B) The 3D model structure of isoform 2 and 3 obtained using I-Tasser (Zhang, 2008) shown to have more simplified structure compared to C1ORF123. [file peerj-06-5377-s010.png]
