# Supplementary material for: Crystal structure and functional analysis of human C1ORF123
Source: PeerJ. 2018 Sep 28;6:e5377. doi: 10.7717/peerj.5377 (PMC6166629; doi:10.7717/peerj.5377)
Supplement: Supplemental Information 11 [file peerj-06-5377-s011.docx]

Supplementary Table 1: The list of rabbit IgG co-immunoprecipitated human proteins from HeLa cells lysate. These proteins were used to eliminate false positive that identified from anti-C1ORF123 co-immunoprecipitated proteins.

| No. | UniProt ID | Accession no | Protein name | Length |
| --- | --- | --- | --- | --- |
| 1 | P04264 | K2C1_HUMAN | Keratin, type II cytoskeletal 1 | 644 |
| 2 | P08708 | RS17_HUMAN | 40S ribosomal protein S17 | 135 |
| 3 | P13645 | K1C10_HUMAN | Keratin, type I cytoskeletal 10 | 584 |
| 4 | P35908 | K22E_HUMAN | Keratin, type II cytoskeletal 2 epidermal | 639 |
| 5 | P35527 | K1C9_HUMAN | Keratin, type I cytoskeletal 9 ( | 623 |
| 6 | P60709 | ACTB_HUMAN | Actin, cytoplasmic 1 | 375 |
| 7 | Q562R1 | ACTBL_HUMAN | Beta-actin-jenis protein 2 | 376 |
| 8 | Q93077 | H2A1C_HUMAN | Histone H2A type 1-C | 130 |
| 9 | Q99878 | H2A1J_HUMAN | Histone H2A type 1-J | 128 |
| 10 | Q16777 | H2A2C_HUMAN | Histone H2A type 2-C | 129 |
| 11 | Q96KK5 | H2A1H_HUMAN | Histone H2A type 1-H | 128 |
| 12 | P0C0S8 | H2A1_HUMAN | Histone H2A type 1 | 130 |
| 13 | P20671 | H2A1D_HUMAN | Histone H2A type 1-D) | 130 |
| 14 | Q7L7L0 | H2A3_HUMAN | Histone H2A type 3 | 130 |
| 15 | Q6FI13 | H2A2A_HUMAN | Histone H2A type 2-A | 130 |
| 16 | P04908 | H2A1B_HUMAN | Histone H2A type 1-B/E | 130 |
| 17 | P63261 | ACTG_HUMAN | Actin, cytoplasmic 2 | 375 |
| 18 | P08670 | VIME_HUMAN | Vimentin | 466 |
| 19 | O14950 | ML12B_HUMAN | Myosin regulatory light chain 12B | 172 |
| 20 | Q9NYL9 | TMOD3_HUMAN | Tropomodulin-3) | 352 |
| 21 | P35579 | MYH9_HUMAN | Myosin-9 | 1960 |
| 22 | P09493 | TPM1_HUMAN | Tropomyosin alpha-1 chain) | 284 |
| 23 | P60660 | MYL6_HUMAN | Myosin light polypeptide 6 | 151 |
| 24 | P68032 | ACTC_HUMAN | Actin, alpha cardiac muscle 1 | 377 |
| 25 | P02768 | ALBU_HUMAN | Serum albumin | 609 |
| 26 | P00761 | TRYP_PIG | Trypsin (EC 3.4.21.4) | 231 |
| 27 | P13647 | K2C5_HUMAN | Keratin, type II cytoskeletal 5 | 590 |
| 28 | P04259 | K2C6B_HUMAN | Keratin, type II cytoskeletal 6B | 564 |
| 29 | Q9BTM1 | H2AJ_HUMAN | Histone H2A.J (H2a/j) | 129 |
| 30 | P02533 | K1C14_HUMAN | Keratin, type I cytoskeletal 14 | 472 |
| 31 | Q9UNX3 | RL26L_HUMAN | 60S ribosomal protein L26-like 1 | 145 |
| 32 | P61254 | RL26_HUMAN | 60S ribosomal protein L26 | 145 |
| 33 | P62899 | RL31_HUMAN | 60S ribosomal protein L31) | 125 |
| 34 | P19013 | K2C4_HUMAN | Keratin, type II cytoskeletal 4 | 534 |
| 35 | P19105 | ML12A_HUMAN | Myosin regulatory light chain 12A | 171 |
| 36 | P35749 | MYH11_HUMAN | Myosin-11 | 1972 |
| 37 | P06753 | TPM3_HUMAN | Tropomyosin alpha-3 chain | 285 |
| 38 | P35580 | MYH10_HUMAN | Myosin-10 | 1976 |
| 39 | P0CG39 | POTEJ_HUMAN | POTE ankyrin domain family member J | 1038 |
